# Structure of the human SAGA coactivator complex

**DOI:** 10.1038/s41594-021-00682-7

**Published:** 2021-11-22

**Authors:** Dominik A. Herbst, Meagan N. Esbin, Robert K. Louder, Claire Dugast-Darzacq, Gina M. Dailey, Qianglin Fang, Xavier Darzacq, Robert Tjian, Eva Nogales

**Affiliations:** 1 California Institute for Quantitative Biology (QB3), University of California, Berkeley, CA USA; 2 Molecular Biophysics and Integrated Bio-Imaging Division, Lawrence Berkeley National Laboratory, Berkeley, CA USA; 3 Department of Molecular and Cell Biology, University of California, Berkeley, CA USA; 4 Biophysics Graduate Group, University of California, Berkeley, CA USA; 5 Howard Hughes Medical Institute, University of California, Berkeley, CA USA; 6 Present Address: Department of Biology, Johns Hopkins University, Baltimore, MD USA; 7 Present Address: School of Public Health, Sun Yat-sen University, Shenzhen, China

**Keywords:** Cryoelectron microscopy, Cryoelectron microscopy, Chromatin, Epigenetics, Transcriptional regulatory elements

## Abstract

The SAGA complex is a regulatory hub involved in gene regulation, chromatin modification, DNA damage repair and signaling. While structures of yeast SAGA (ySAGA) have been reported, there are noteworthy functional and compositional differences for this complex in metazoans. Here we present the cryogenic-electron microscopy (cryo-EM) structure of human SAGA (hSAGA) and show how the arrangement of distinct structural elements results in a globally divergent organization from that of yeast, with a different interface tethering the core module to the TRRAP subunit, resulting in a dramatically altered geometry of functional elements and with the integration of a metazoan-specific splicing module. Our hSAGA structure reveals the presence of an inositol hexakisphosphate (InsP_6_) binding site in TRRAP and an unusual property of its pseudo-(Ψ)PIKK. Finally, we map human disease mutations, thus providing the needed framework for structure-guided drug design of this important therapeutic target for human developmental diseases and cancer.

## Abstract (web summary)

The cryo-EM structure of the human SAGA coactivator complex reveals high-resolution details of the core and TRRAP modules, providing insights on a metazoan-specific architecture and the structural basis for incorporation of the splicing module in mammalian cells.

## Main

**Fig. 1:**
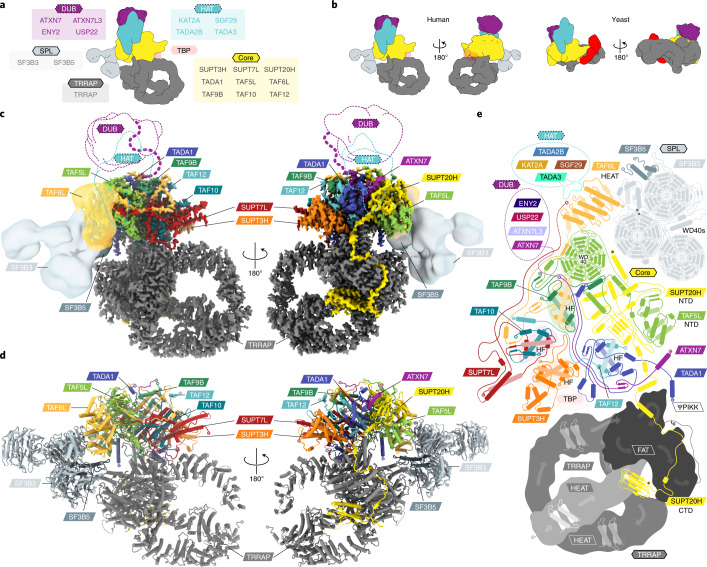
The modular architecture of hSAGA. **a**, Schematic organization of hSAGA subunits into modules (boxes). The putative TBP binding region (based on ySAGA) is indicated as translucent red shape, because evidence for TBP binding by hSAGA is still lacking. **b**, Comparison of hSAGA (left) and ySAGA^5^ (right) after superposition on the core module (Supplementary Fig. 1). Yeast modules are colored according to their human homolog. The yeast TBP binding module is shown in red. Viewing orientations in all other figures are indicated by these schemes. **c**, Hybrid map of hSAGA. The high-resolution cryo-EM map (core and TRRAP) (contoured at 7.0*σ*) and the negative stain map for the TAF6L HEAT domain and the SPL module are shown (contoured at 6.1*σ*). The expected DUB and HAT locations are indicated in dashed lines. Subunits are colored as indicated by the labels. **d**, Atomic model of hSAGA. **e**, Topology map of hSAGA subunits grouped by modules (not drawn to scale). Color schemes are consistent throughout all figures.

**Extended Data Fig. 1:**
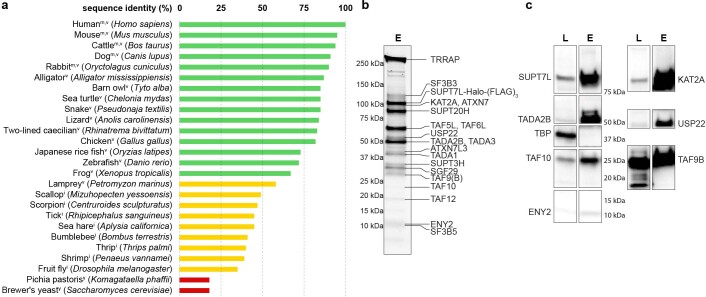
Conservation and Purification of hSAGA. **a**, Average sequence conservation of SAGA subunits in metazoan and yeast (relates to Supplementary Table 1). Indices indicate classification into mammals (m), vertebrate (v, green), invertebrate (i, yellow), and yeast (y, red). **b**. 4-20% gradient SDS-PAGE gel stained with Flamingo fluorescent protein stain (BioRad) of the hSAGA FLAG elution (E) with subunits labeled based on their predicted molecular weight. **c**. Western blot probing for DUB, HAT, and core subunits to verify the presence of these modules in the sample used for grid preparation. TBP did not co-purify with hSAGA. The lysate lane (L) corresponds to 0.0004% of the total input and the right lane (hSAGA Elution, E) corresponds to 4.65% of the final elution. Blots were cropped. Experiments in b and c were repeated twice with similar results. Source data

Transcription of protein coding genes depends on the essential coactivators TFIID and SAGA (Spt-Ada-Gcn5 acetyltransferase)^1,2^. SAGA regulates gene expression by interacting with enhancer-bound activators, recruiting the transcriptional machinery and modifying promoter-proximal chromatin^1^ and is known to be involved also in DNA damage repair and signaling^3^. Previous studies focused primarily on yeast SAGA (ySAGA), and the first structures of the 19-subunit ySAGA were proposed to be also representative of human SAGA (hSAGA)^4,5^. However, hSAGA has noticeable functional and compositional differences, indicative of a divergent architecture, that integrates metazoan-specific U2 splicing subunits and lacks the essential subunit for TATA-box binding protein (TBP) binding in yeast^6^. Human SAGA is a 20-subunit, 1.4-MDa complex with five functional modules (Fig. 1a): a scaffolding core that includes TBP-associated factors (TAFs); a TRRAP (Transformation/Transcription domain Associated Protein) containing a phosphoinositide-3-kinase (PI3K)-related pseudoprotein kinase (ΨPIKK); a histone acetyltransferase (HAT); a deubiquitinase (DUB) and a metazoan-specific splicing (SPL) module^7^. The recent structural characterization of the 19-subunit *Saccharomyces* *cerevisiae* and *Komagataella* *phaffii* ySAGA at 3.8–3.9 Å resolution provided insights into the histone-fold core, TBP binding and overall ySAGA architecture^4,5^, with the HAT and DUB domains being flexibly attached to the core. While vertebrate SAGA is highly conserved (roughly 95–58% sequence identity), the conservation with yeast drops dramatically (roughly 18% sequence identity) (Extended Data Fig. 1a and Supplementary Table 1), and numerous domain insertions, deletions and gene duplications have led to subfunctionalization of hSAGA subunits^8^ and to hSAGA being essential for development in vertebrates (in contrast, ySAGA is not essential for viability)^3,7^. The compositional and functional differences between the yeast and human complexes hinted at possible structural differences and led us to examine the structure of hSAGA using cryogenic-electron microscopy (cryo-EM).

## Results

### Architecture of human SAGA

**Extended Data Fig. 2:**
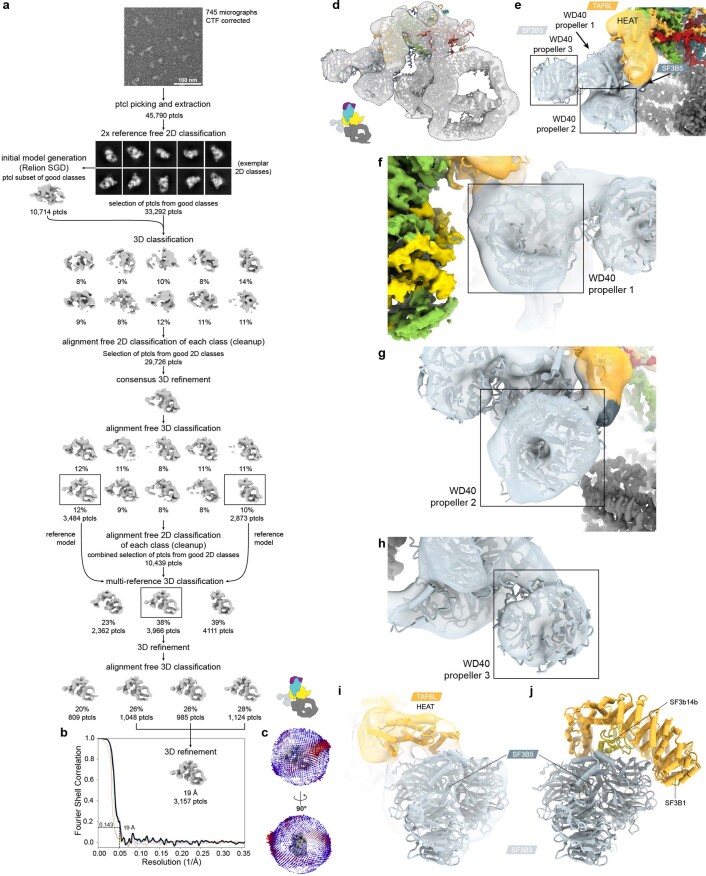
Negative stain processing scheme and model fit. A representative section of one of 745 micrographs is shown. After initial 2D classification, particles (ptcls) from the best classes were used for initial model generation. The data was cleaned up by 3D classification followed by alignment-free 2D classification. Particles from all good classes were subjected to a consensus 3D refinement followed by alignment-free 3D classification. All except for one class revealed fuzzy density for the TAF6L HEAT and SPL region. Subsequently, all classes were cleaned up individually by alignment-free 2D classification and combined in a multi-reference classification using the two best models with and without TAF6L HEAT and SPL region. The best class including this region was subjected to 3D refinement, alignment-free 3D classification, and to a final refinement using particles of the class combination that yielded highest resolution. **b**. Final map and FSC plot. **c**. Angular distribution. **d**. Final map (contoured at 4.9 σ) and rigid body fit of SF3B3/SF3B5 (from PDB: 5IFE), a homology model of the TAF6L HEAT domain, and the cryo-EM structure from this study. **e-h**. Close-up view on the SPL module region of the hybrid map shown in Fig. 1c. The rigid-body fit is shown with translucent map surfaces. All domains fit precisely in the negative stain density, which shows clear central holes for the three WD40 propellers (f-h) of SF3B3. **i, j**. Comparison of the SF3B3/SF3B5 integration in hSAGA and the SF3b complex. **i**. The SPL module (SF3B3 and SF3B5 subunits) binds to the concave surface of the TAF6L HEAT domain. The negative stain map of hSAGA is shown in translucent white (contoured at 4.8 σ). **j**. Crystal structure of the SF3b complex^10^ (PDB: 5IFE). The TAF6L HEAT domain of hSAGA is replaced by the SF3B1 HEAT repeat domain in the SF3b complex. Both domains share an overlapping binding region on the SF3B5 surface.

**Extended Data Fig. 3:**
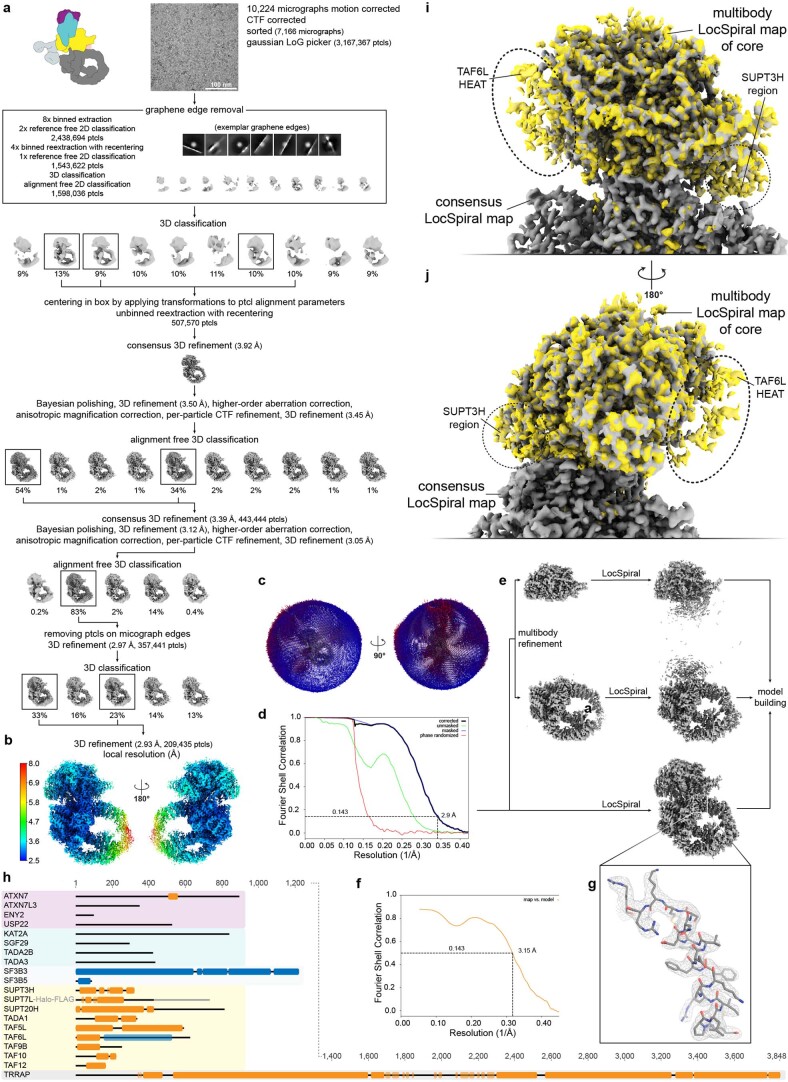
Cryo-EM processing and model building. A representative section of one of 10,224 micrographs is shown. Graphene oxide (GO) edges were removed in cycles of initial 2D and one 3D classification. The negative stain reconstruction (see Extended Data Fig. 2) was used as initial model. 3D classes were centered in the box by applying a coordinate transformation to the alignment parameters, and unbinned particles were re-extracted with recentering. Particles were filtered for high-resolution features in cycles of 3D refinement, classification, Bayesian polishing, and CTF refinement as indicated. **b**. Postprocessed map (B-sharpened with -51.1 Å^2^, contoured at 4.9 σ) with local resolution. **c**. angular distribution. **d**. Fourier Shell Correlation (FSC). **e**. Multibody refinement improved map quality, but not the overall resolution. Considerable improvement of map quality was achieved by filtering with LocSpiral^50^. The model for the core and TRRAP was built into the LocSpiral filtered maps of the multibody refinement. The interface between these regions was built using the full map and used for model refinement. Refinement against the postprocessed map (**b**) resulted in the same model, with virtually identical statistics and an all-atom r.m.s.d. of 0.400 Å. Maps are contoured at (regular/LocSpiral): Core 11.2 σ/9.2 σ, TRRAP 7.9 σ/9.0 σ, full 6.9 σ. **f**. Map vs. model FSC using the postprocessed map shown in b. **g**. The refined map shows well defined secondary structure elements and side chains (contoured at 9.0 σ). **h**. Model-sequence coverage. Sequences of all subunits are indicated as horizontal lines (black) and modeled regions as overlaying boxes (orange: visualized by cryo-EM; blue: visualized only by negative stain; translucent: regions with unclear register assignment (unknown, UNKs)). **i, j**. The LocSpiral filtered multibody map of the core reveals additional density corresponding to the poorly ordered TAF6L HEAT domain, and to SUPT3H in the cleft between the core and TRRAP module (both contoured at 5.9 σ).

For our structural studies, we purified intact, endogeneous hSAGA from HeLa cells (Methods). The presence of all 20 hSAGA subunits was validated using western blotting and mass spectrometry (Extended Data Fig. 1b,c, Supplementary Table 2 and Methods). Single-particle negative stain (Table 1 and Extended Data Fig. 2) showed the presence of a central core domain sitting atop the distinct cradle-shaped TRRAP module, with a Y-shaped density flexibly tethered to the core domain proximal to the TRRAP cradle. Our negative stain reconstruction at 19 Å resolution (Extended Data Fig. 2d) already revealed clear architectural differences with respect to the ySAGA complexes^4,5^ (Fig. 1a,b). Using cryo-EM we then obtained a reconstruction (overall resolution of 2.9 Å; Fig. 1c, Table 1 and Extended Data Fig. 3a–g) that allowed us to build an atomic model for the best ordered regions of hSAGA: the core module, consisting of TAF5L, SUPT20H, seven histone-fold-containing subunits (TAF6L, TAF9B, TAF10, TAF12, SUPT7L, TADA1 and SUPT3H) and the DUB anchor subunit ATXN7, and the large TRRAP subunit that consists of a circular HEAT (Huntingtin, elongation factor 3 (EF3), protein phosphatase 2A (PP2A), and the yeast kinase TOR1) repeat cradle, FAT (FRAP, ATM and TRRAP) and pseudo-(Ψ)PIKK domains (Fig. 1d,e, Extended Data Fig. 3 and Supplementary Video 1).

**Table 1:** Cryo-EM data collection and refinement statistics

	Cryo-EM (EMDB-23027) (PDB 7KTR)	Negative stain (EMDB-23028) (PDB 7KTS)
**Data collection and processing**		
Microscope	FEI Titan Krios G2	FEI Technai F20
Camera	Gatan K3 Summit (super resolution)	Gatan UltraScan4000
Voltage (keV)	300	120
Magnification	64,000	80,000
Defocus range (μm)	0.9–3.4	0.4–3.9
Micrographs/videos	10,224	1
Frames per video	50	NA
Pixel size (Å)	1.187	1.4
Total dose (e−/Å^−2^)	50	35
Symmetry imposed	C1	C1
Particles initial/final	3,167,367/357,441	47,790/3,157
Map resolution (Å)	2.9	19
FSC threshold	0.143	0.143
Map resolution range (Å)	2.5–9.0	NA
**Model refinements**		
Initial model used (PDB code)	6F3T, de novo	7KTR, 5IFE
Method	real space, adp	rigid body^a^
Model resolution (Å)	3.0	NA
FSC threshold	0.5	NA
Model resolution range (Å)	2.3–25.4	NA
Map-to-model cross-correlation	0.80	0.52
Map sharpening *B* factor (Å^2^)	NA^b^	−1,200
Model composition		
Nonhydrogen atoms	40,337	51,173
Protein residues	5,169	6,632
Ligands	1	0
Mean model *B* factors (Å^2^)		
Protein	73.6	not refined
Ligand	66.6	not refined
R.m.s. deviations		
Bond lengths (Å)	0.004	0.005
Bond angles (°)	0.62	0.99
Validation		
MolProbity score	1.50	1.62
All-atom clashscore	3.42	4.79
Rotamer outliers (%)	0.02	0.02
C-beta deviations	0	0
Ramachandran plot		
Favored (%)	94.72	94.57
Allowed (%)	5.24	5.38
Disallowed (%)	0.04	0.05
Rama-*Z* score, whole (r.m.s.)	0.16 (0.12)	−0.35 (0.11)

Cryo-EM data collection and refinement statistics. ^a^Three bodies were fit comprising the SF3B3/SF3B5 subunits of SF3b (PDB 5IFE), a homology model based on human TAF6 (PDB 6MZL) and the cryo-EM structure of hSAGA. ^b^LocSpiral filtered: NA, not applicable; adp, atomic displacement parameters.

Due to the flexible nature of the region connecting the Y-shaped density, this region could not be resolved in the high-resolution cryo-EM reconstruction (Extended Data Fig. 3i,j). However, by superposing the common elements with the negative stain structure and following the main chain density for TAF6L, we were able to unambiguously assign this region to the metazoan-specific SPL module (Fig. 1c,d). We were able to dock with high precision a homology model of the TAF6L HEAT domain^9^ as well as the SF3B3/SF3B5 subunits of the SF3b crystal structure^10^ into our 19 Å map (Extended Data Fig. 2e–h).

**Fig. 2:**
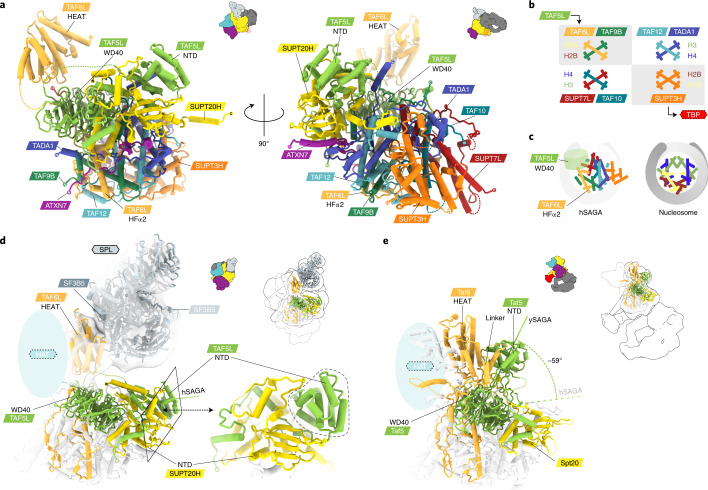
Organization of the core and integration of the SPL module. **a**, Atomic model of hSAGA core. **b**, Schematic comparison of the histone-fold core organization in hSAGA with the nucleosome. Histone-fold dimers are grouped in boxes, colored by hSAGA subunits (above/below). Corresponding histones are indicated on the side, as well as the contact with the TAF5L WD40 propeller and the potential interaction with TBP below/above. **c**, Schematic of the relative locations of the distorted histone-fold octamer helices in the hSAGA core and in the nucleosome. **d**, The (concave) surface of the TAF6L HEAT domain tethers the SPL module in hSAGA (translucent EM contoured at 6.1*σ*). The SUPT20H NTD latches the TAF5L NTD in place (a close-up on the right). **e**, In ySAGA, the Taf5 NTD is rotated −59° and occupies the corresponding Taf6 HEAT domain surface (PDB 6T9K). The location of the HAT modules in **d** and **e** is indicated by translucent blue ovals and location of the depicted regions in the context of the complete complexes are outlined at the top right.

**Extended Data Fig. 4:**
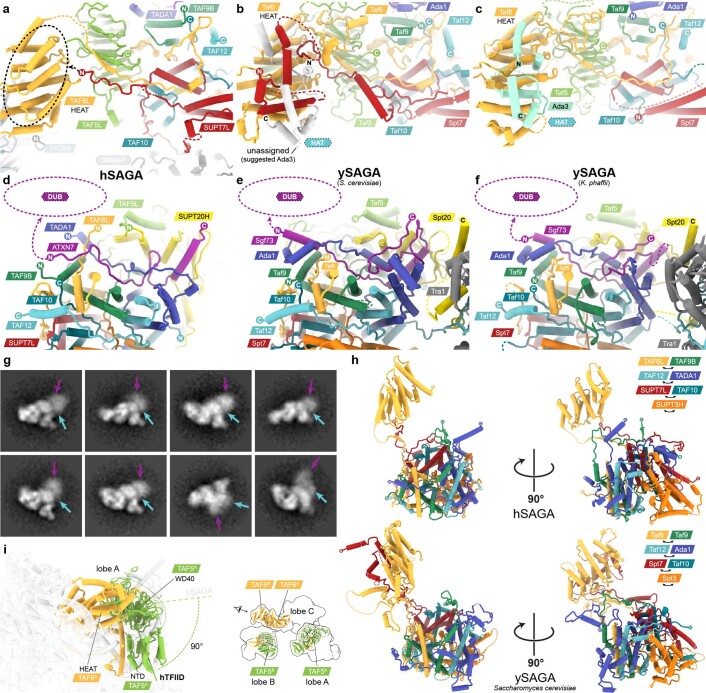
The core module and tethering of its peripheral modules in human and yeast. **a-c**. Tethering of the HAT module: **a**. The N-terminus of SUPT7L runs parallel to the TAF6L linker, which connects to the HEAT domain, along the surface of the core and ends with its N-terminus in close proximity to the HEAT domain. **b**. In *Saccharomyces cerevisiae*^4^ (PDB: 6T9I), the Spt7 linker further extends towards the convex surface of the TAF6L HEAT domain and interacts with an unassigned region. **c**. The same region in *Komagataella phaffii*^5^ was assigned as the Ada3 subunit of the HAT module (PDB: 6TBM). The similar location of the SUPT7L N-terminus suggests a similar interaction and connectivity for the HAT module in hSAGA. **d-f**. Tethering the DUB module. **d**. The ATXN7 subunit of the core and the DUB module (Sgf73 in yeast) is similarly integrated into the core module as in ySAGA (**e**, **f**), suggesting a similar relative attachment of the human DUB. **g**. GraFix^54^ crosslinked negative stain class averages revealed extra density at the anticipated locations for the HAT (cyan arrow) and DUB (purple arrow) modules. **h**. The core of hSAGA and ySAGA (PDB: 6T9K) builds on common HF elements. Only HF containing subunits are shown. Architectural differences are created by local variations outside of the HFs. HF dimerization is indicated by arrows below the subunit labels. **i**. Comparison of TAF5 and TAF6 architecture within lobe A of human TFIID (canonical state^9^, PDB: 6MZL). TFIID contains two copies of TAF5 and TAF6, with one TAF5 located in lobe A (TAF5^A^) and the other one in lobe B (TAF5^B^), and with the two TAF6 HEAT domains (TAF6^A^, TAF6^B^) in lobe C (shown on the right). Compared to hSAGA, the TFIID TAF6 HEAT domains are differently arranged relative to TAF5, and they act to bridge lobes B and C. The TFIID TAF5 NTD is rotated by 90°, leading to a divergent architecture.

While all hSAGA HAT and DUB subunits were confirmed in our sample, they were not resolved in our structural analysis, either due to flexible tethering or a more dynamic and labile attachment, consistent with the low resolution and flexibility described for these modules in the yeast complex^4,5^. By comparing the positions of the HAT-tethering subunits TAF6L/SUPT7L and the integration of the DUB subunit ATXN7 in the core (Fig. 2a) with their respective counterparts in ySAGA, we anticipate similar general positions for these modules in hSAGA (Extended Data Fig. 4a–f). Moreover, very weak density, visible only in some class averages after gradient crosslinking (Extended Data Fig. 4g and Methods), likely correspond to the HAT and DUB domains, indicating a flexible or dynamic connection to the complex at the expected positions.

### Core module structure and tethering of the SPL module

The structure of hSAGA is organized around the nine-subunit core module (Figs. 1a,c–e and 2a), in which the seven subunits contain histone folds (SUPT3H containing two) assemble into a distorted pseudo-octamer (Fig. 2b,c), as also observed in ySAGA^4,5^ (Extended Data Fig. 4h), as well as in human and yeast TFIID^9,11,12^. The distortion from the symmetric nucleosomal octamer creates a gap that is filled by the TAF5L WD40 propeller, which centrally binds to helix ∝2 of the TAF6L histone fold (Fig. 2a,c). The periphery of the core is organized by the C-terminal TAF6L HEAT repeat domain, which connects the SPL module on its concave side (Figs. 1c and 2d) and probably the HAT module on its convex side (Extended Data Fig. 4a–c). Such connections are consistent with yeast two-hybrid assays of *Drosophila* homologs, which suggested interactions between SF3B3 and SF3B5 (SPL), SGF29 (HAT) and SUPT7L (Core)^13^. The SF3B3 subunit contains three WD40 propellers and tethers the SPL module via propeller one and two to the TAF6L HEAT repeats (Fig. 2d and Extended Data Fig. 2e,i). Of note, in ySAGA, the corresponding interface on the Taf6 HEAT repeat is blocked by the Taf5 N-terminal domain (NTD) (Fig. 2e). This domain is rotated −59° relative to the human TAF5L NTD, which in hSAGA is latched in place by the SUPT20H NTD (Fig. 2d).

### SUPT20H as a latch and binding of InsP_6_

**Fig. 3:**
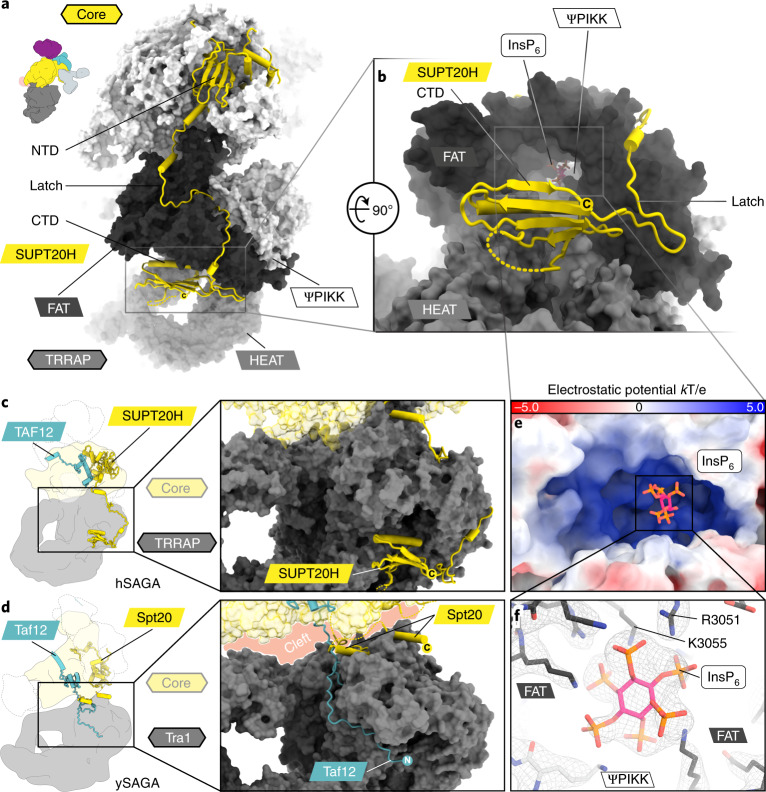
SUPT20H structure and interactions. **a**, SUPT20H contains an NTD in the core and a CTD in the TRRAP module, that are connected via a linker (‘latch’) that runs along a surface groove around the TRRAP FAT domain. TRRAP domains are colored in different shades of gray. **b**, The CTD is located at the entrance to a positively charged tunnel below the FAT domain. **c**,**d**, Schematic and close-up view of hSAGA (**c**) and ySAGA (**d**, PDB 6T9I), showing the different interactions of SUPT20H/Spt20 and TAF12/Taf12 with TRRAP/Tra1. The cleft between the Tra1 and core module is indicated in light red (**d**). **e**, Electrostatic surface potential representation of the positively charged tunnel (**b**) with InsP_6_ (**f**) (Extended Data Fig. 6). **f**, LocSpiral filtered multibody map (contoured at 11*σ*) showing the InsP_6_ site. Carbon atoms are colored by TRRAP domain or pink for InsP_6_, red (oxygen), blue (nitrogen) or orange (phosphorus).

**Extended Data Fig. 5:**
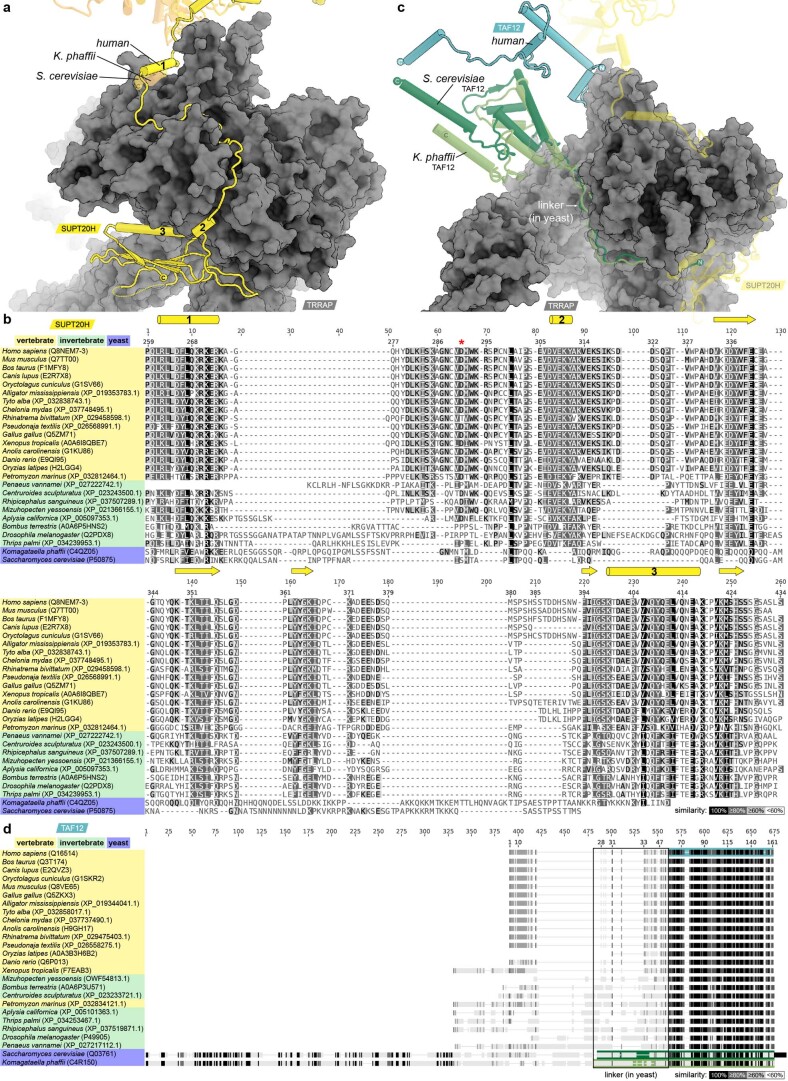
Human versus yeast interactions between TRRAP/Tra1, SUPT20/Spt20 and TAF12/Taf12. **a**. Location of the SUPT20H/Spt20 C-terminal region after superposition of human TRRAP and yeast Tra1. The C-terminal helix of yeast Spt20 aligns with helix one of the SUPT20H linker. **b**. The sequence alignment of the SUPT20H/Spt20 C-terminal regions for 24 metazoan (SUPT20H) and two yeast (Spt20) species shows that the SUPT20H CTD is highly conserved in vertebrates, while it does not appear to exist in yeast. Secondary structure elements are indicated above the alignment. *: D291 forms a salt bridge with TRRAP R3746 (Fig. 5f). Vertebrate and invertebrate sequences were pre-aligned to human SUPT20H, regions corresponding to the structured part in **a** were extracted and realigned with the yeast sequences corresponding to the region from helix 1 in **a** to the C-terminus. **c**. Relative location of the TAF12 N-terminal region, based on the superposition shown in **a**. In yeast, an N-terminal linker of Taf12 wraps around the inside of the Tra1 FAT domain, while human TAF12 contacts TRRAP in a different location. The structured N-terminus of yeast Taf12 is located in the same relative position as the human SUPT20H CTD. **d**. Zoomed-out sequence alignment of 24 metazoan and two yeast TAF12/Taf12 subunits. Structured regions are colored as in **c** and the region corresponding to the linker in yeast is indicated. Aligned regions in **b** and **d** are colored by similarity in gray scale (annotated in **d**). In yeast, Taf12 contains a considerably longer N-terminus that appears to be unique to yeast. Sequences are labeled as: Scientific organism name (Uniprot or NCBI accession code). The organism selection corresponds to Extended Data Fig. 1a and Supplementary Table 1.

SUPT20H forms the largest interface with the rest of the complex (approximately 12,000 Å^2^) and acts as a clamp-like scaffold within hSAGA (Fig. 3a), supporting its central role in complex assembly and module association^14,15^. Our structure shows how SUPT20H tethers the DUB anchor ATXN7 to the core (Fig. 2a and Extended Data Fig. 4d). In addition to its crucial role in latching away the TAF5L NTD, thus allowing incorporation of the SPL module (described above), SUPT20H also makes extensive contacts between the core and TRRAP module that contribute to create an architecture very different from that of ySAGA. The SUPT20H NTD connects to a long linker, ‘the latch’ (Fig. 3a), missing in yeast, that wraps along the surface of the core, around the TRRAP FAT domain and terminates in the cleft below the FAT and central TRRAP HEAT repeats with a previously unpredicted C-terminal domain (CTD) (Fig. 3b). The CTD folds into a five-stranded antiparallel beta-sheet with an alpha-helix parallel to the sheet that connects the two C-terminal outer strands (Figs. 1c–e and 3b). The closest structural homolog is the Spt6 SH2 domain of *Candida glabrata*^16^ (2.20 Å C_∝_-r.m.s.d. over 49 residues). Neither the SUPT20H latch, nor its CTD are conserved in ySAGA (Fig. 3c,d and Extended Data Fig. 5a,b). On the other hand, the N terminus of ySAGA Taf12, lacking in the human homolog, emerges from a location similar to that of the SUPT20H CTD and wraps around the opposite side of the Tra1 FAT domain (Fig. 3c,d). Metazoan TAF12s have a much shorter N terminus and contact TRRAP at a different location (Extended Data Fig. 5c,d).

**Extended Data Fig. 6:**
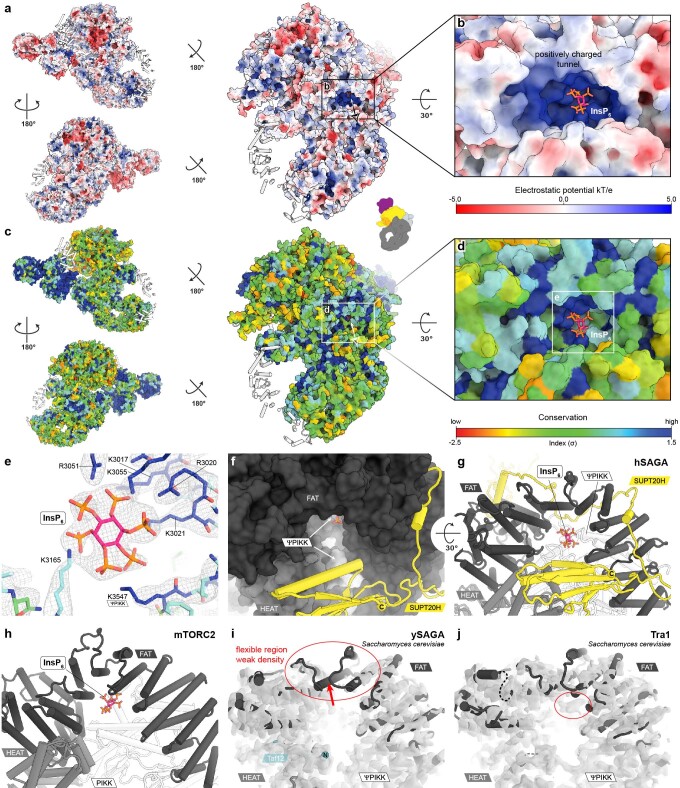
Electrostatic surface, conservation, and binding of inositol hexakisphosphate (InsP6) to TRRAP. **a**. Electrostatic surface representation of hSAGA shown from three different views. Only regions with all-atom models have been included. Regions of lower sequence assignment confidence (unknown, UNKs) were excluded from the calculation and are shown as white cartoon. A highly positive charged tunnel between the FAT, HEAT, and ΨPIKK domains of TRRAP and SUPT20H is indicated. **b**. Close-up view of the InsP_6_ binding pocket within this tunnel. **c**. Same views as in **a** colored by sequence conservation (white cartoon as in **a**). **d**. Close-up view of the InsP_6_ binding pocket. InsP_6_ (shown in stick representation) is bound by a half-ring of highly conserved residues. **e**. Close-up view of the atomic model and the LocSpiral filtered multibody map (contoured at 11 σ) of TRRAP. The view corresponds to a back view of Fig. 3f (rotated 180°). Residues involved in InsP_6_ binding are indicated. All labeled residues are part of the TRRAP FAT domain except for K3547 (within ΨPIKK). Atoms are colored by conservation (carbon, see panel d), pink (carbon of InsP_6_), red (oxygen), blue (nitrogen), or orange (phosphorus). **f**. View as indicated by boxes in a and c (colored by domains and subunits). **g, h**. Comparison of InsP_6_ binding in hSAGA and in human mTORC2^31^ shown in cartoon representation. In both complexes InsP_6_ binds in a similar location between the FAT and pseudo- and kinase domains of hSAGA and mTORC2, respectively. **i**. In the ySAGA complex structures (here PDB: 6T9J) the central part of the FAT domain is poorly resolved (red circle) and presumably flexible (translucent map contoured at 11 σ). This region contains residues (red arrow) involved in InsP_6_ binding in hSAGA (*for example* R3051 and K3055, see panel e). **j**. Unattributed density (red circle) in the earlier determined structure of isolated Tra1 (PDB: 5OJS, translucent map contoured at 7.5 σ).

The CTD location of SUPT20H resembles a lid at the entrance of a positively charged tunnel below the FAT domain that is conserved in metazoans (Fig. 3e and Extended Data Fig. 6a–d). In a side pocket of this tunnel and bound to highly conserved residues of the FAT and ΨPIKK domains, our structure shows clear density for the metabolite inositol hexakisphosphate (InsP_6_), which copurified with hSAGA (Fig. 3b,e,f and Extended Data Fig. 6e–j).

### TRRAP structure and interactions with the core module

**Fig. 4:**
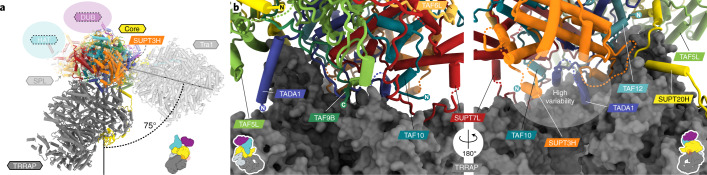
Distinct TRRAP tethering in hSAGA. **a**, Superposition of the hSAGA and ySAGA (PDB 6T9I) (translucent) models on the core. Other module locations are indicated by colored ovals. **b**, Close-up views of the hSAGA TRRAP–core interface. A region of high variability is indicated (Extended Data Fig. 8b,c).

**Extended Data Fig. 7:**
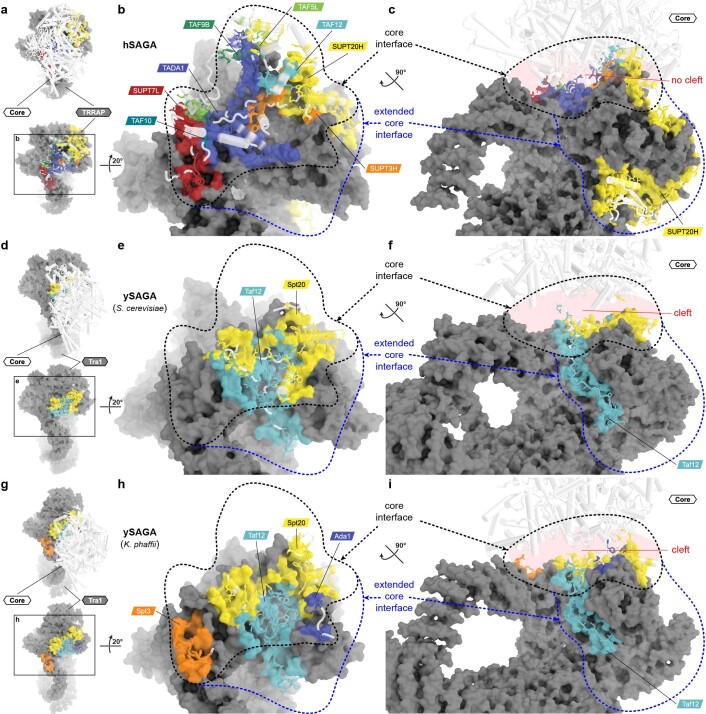
Comparison of the core-TRRAP/Tra1 interface in human and yeast. **a**. Top view on the hSAGA core (white cartoon) and TRRAP module (gray surface). Parts of the core not in direct proximity to the interface have been removed in the lower panel. Interfacing residues on the TRRAP surface are colored based on their closest core subunit. Interfacing residues of core subunits are shown in stick representation and colored by subunit. **b**. Magnified region from the box in a. The interface corresponding to the footprint of the core on the TRRAP surface is indicated with a black dashed outline (core interface). The interface created by extensions of core subunits that wrap around the TRRAP is indicated with a blue dashed outline (extended core interface). In hSAGA the latch and SUPT20H CTD (residues 274-428) contribute to the extended core interface. **c**. Side view of the core and extended interface with the complete core shown as white cartoon. In contrast to ySAGA (f, i), hSAGA has no cleft between the core and TRRAP modules. **d**,**f**. Same view as in a-c for *S. cerevisiae* ySAGA (PDB: 6T9I)^4^. **g**,**h**. Same view as in a-c for *K. phaffii* ySAGA (PDB: 6TB4)^5^. The colored surface regions on TRRAP/Tra1 correspond to the colors of the contacting core subunits and thus show the different participation of core subunits to the interface. **f, i**. In both ySAGA structures the core and Tra1 modules are separated by a cleft.

The TRRAP subunit, like the yeast Tra1, has a characteristic tripartite HEAT repeat organization, consisting of a central N-terminal repeat and a circular cradle, followed by a FAT domain and a ΨPIKK (Fig. 1c–e) (the Tra1 and TRRAP subunits are shared with the yeast NuA4 complex and its human counterpart, TIP60, respectively)^4–6,17^. Compared to ySAGA, hSAGA exhibits a dramatically different TRRAP–core interface that leads to a relative rotation of 75° of TRRAP/Tra1 with respect to the core and SUPT3H/Spt3 (Fig. 4a). While the approximate region of the interface is similar on TRRAP and Tra1, the region on the core contributing to the interface is dramatically different for yeast and human complexes. In hSAGA, all core subunits except for TAF6L and ATXN7 are involved in the TRRAP–core interface (Fig. 4b and Extended Data Fig. 7a–c), as compared to a limited number in yeast (Extended Data Fig. 7d–i). In ySAGA, the core subunits Spt20 and Taf12 form local interactions on the Tra1 surface and are connected to the core by flexible linkers that span a large cleft between the modules (Fig. 3d and Extended Data Fig. 7f,i). Such a cleft does not exist in hSAGA (Extended Data Fig. 7c) and presumably leads to the increased flexibility observed between the core and Tra1 in yeast^18,19^. While the main TRRAP–/Tra1–core interfaces, corresponding to the core’s footprint on TRRAP or Tra1 (Fig. 4b and Extended Data Fig. 7b,c,e,f,h,i), are of a similar size (roughly 3,500 Å^2^), both complexes rely on additional stabilization by unique extensions of either Taf12 in yeast (Fig. 3d, Extended Data Figs. 5c and 7f,i and Supplementary Table 3) or SUPT20H in human that form interfaces with different regions on Tra1 and TRRAP, respectively (Fig. 3c and Extended Data Figs. 5a and 7c). In hSAGA, the SUPT20H extension doubles the total interface (to 7,073 Å^2^), which is ultimately 64% larger than that of ySAGA (Supplementary Table 3).

## Discussion

### Local variations in the core enable a divergent architecture

**Extended Data Fig. 8:**
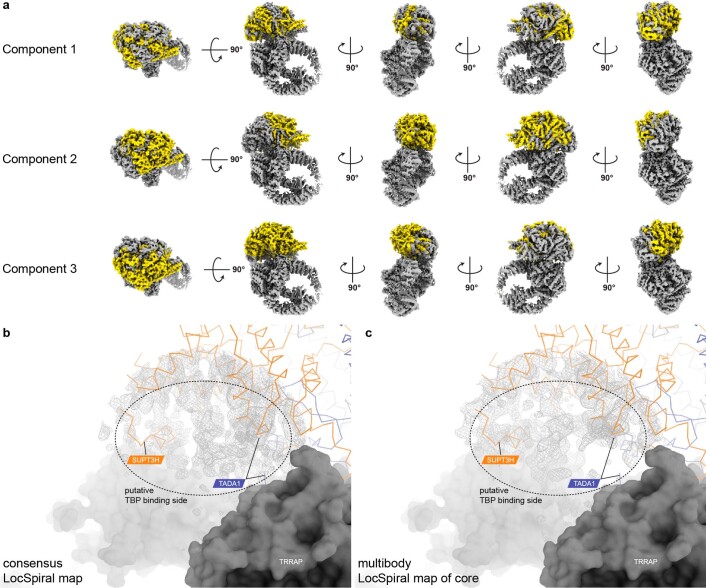
Conformational variability in hSAGA between the core and TRRAP domains. **a**. Principal component analysis of the multibody refinement reveals several tilting and swiveling motions between the core and TRRAP module. **b, c**. The cleft between SUPT3H, TADA1 (colored ribbon representation), and TRRAP (surface representation) reveals highly variable density, shown in grey mesh with a radius of 20 Å, in the LocSpiral filtered consensus maps of the core (b, contoured at 6 σ) and the multibody (c, contoured at 9.2 σ). Other core subunits are indicated in light grey ribbon. A region of high variability is shown with the dashed oval (Relates to white oval in Fig. 4b).

Our study revealed that local variations, such as the repositioning of the TAF5L NTD and different interactions of SUPT20H and TAF12 on the TRRAP surface, result in very different interfaces between the structurally conserved cores of ySAGA and hSAGA with the Tra1 and TRRAP subunits, respectively. Consequently, this nonconserved geometry positions functional elements in the core and the activator-binding subunit in totally different relative orientations. While the hSAGA TRRAP–core interface is not entirely rigid (Extended Data Fig. 8a), a potential transition between the observed yeast and human conformations, is extremely unlikely. The yeast Taf12 N terminus and Spt20 C terminus form local interactions on the surface of Tra1 beyond the cleft and are likely to move with it as one body. Rearrangement from the yeast to the human conformation would require unfolding of these elements on Tra1 or of parts of the Taf12 histone fold. Similarly, a transition from the human to the yeast conformation would require unfolding of SUPT20H NTD elements that are involved in TAF5L NTD binding. Such a transition far exceeds the conformational space that these modules appear to be capable of exploring. Within the NuA4 complex, Tra1 has been shown to connect to the rest of the complex using a similar, albeit larger interface region as in ySAGA^20^, suggesting that the newly defined TRRAP interface in hSAGA might also be relevant for TRRAP incorporation into the related metazoan TIP60 complex.

### Human SAGA and TBP

Analysis of our cryo-EM data (Methods) revealed heterogeneity that suggests alternative main chain conformations in the cleft between TRRAP and SUPT3H/SUPT7L/TADA1, which includes the region where TBP is bound by Spt3 (SUPT3H homolog) and the yeast-specific Spt8 (ref. ^5^) (Fig. 1e and Extended Data Figs. 3i,j and 8). We could not observe density for TBP, even when it was added in excess to the purified hSAGA (Methods), in contrast with the observations for the yeast complex^5^, highlighting another distinct difference between these complexes. The lack of a stable TBP–hSAGA complex may either indicate that hSAGA does not bind TBP at all, or, together with the observed electron microscopy (EM) heterogeneity, might indicate a highly dynamic or regulated mode of TBP binding, unlike that for the TFIID or ySAGA complex, that may require stabilization by additional factors. Metazoans lack a homolog of the yeast subunit Spt8, which is sufficient for TBP binding on its own, whereas Spt3 is not^21^. On the other hand, the transcription factor c-Myc has been shown to interact with TBP^22^ as well as TRRAP^23,24^ via nonoverlapping regions, suggesting the intriguing possibility that activators could play a role in TBP recruitment to metazoan SAGA. DNA binding by ySAGA-bound TBP was shown to be sterically hindered by Tra1 (ref. ^5^). However, due to the distinct tethering of TRRAP in hSAGA, any interaction of TBP with hSAGA could have different consequences on TBP–DNA binding.

### Metazoan incorporation of a SPL module

Comparison of our structure with that of ySAGA reveals a crucial rearrangement of the TAF5L NTD within the core. The lack of a stabilizing TAF6L HEAT–TAF5L NTD interaction probably contributes to increased flexibility of the TAF6L HEAT repeat domain, a critical platform for HAT and SPL module integration. Furthermore, the local repositioning of the TAF5L NTD exposes the TAF6L interface to allow for SPL module incorporation in hSAGA. The position of the TAF5 NTD is also dramatically different from that observed in Lobe A and Lobe B of TFIID, making this domain a crucial marker for the divergent architectures of TAF-containing complexes^9,11^ (Extended Data Fig. 4i). While our EM structure revealed the site of incorporation of the SPL module, very little is yet known about its function or how its components partition between SAGA and the U2 small nuclear ribonucleoprotein. It has been proposed that SF3B3 incorporation into hSAGA may play a role in ultraviolet (UV) -damaged DNA binding and repair^25^, but contradictory results argued that the structurally related subunit DDB1, which we did not observe in our sample, is the one that recognizes UV-damaged DNA in the context of hSAGA (Supplementary Table 2)^26^. The SPL module subunits, SF3B3 and SF3B5, are shared with the metazoan spliceosomal SF3b core complex within the U2 small nuclear ribonucleoprotein. Our structure shows that they are tethered to the rest of the hSAGA complex in a similar way as they are in the spliceosomal SF3b complex^27^. In hSAGA, SF3B3 binds to the HEAT repeat domain of TAF6L, while SF3B3 binds to the HEAT solenoid of SF3B1 in the SF3b complex^10^, and they do so using an overlapping interface (Extended Data Fig. 2i,j). Therefore, SF3B3/SF3B5 cannot be simultaneously incorporated into hSAGA and the SF3b SPL complex.

### Pseudo-kinase active site in TRRAP

**Extended Data Fig. 9:**
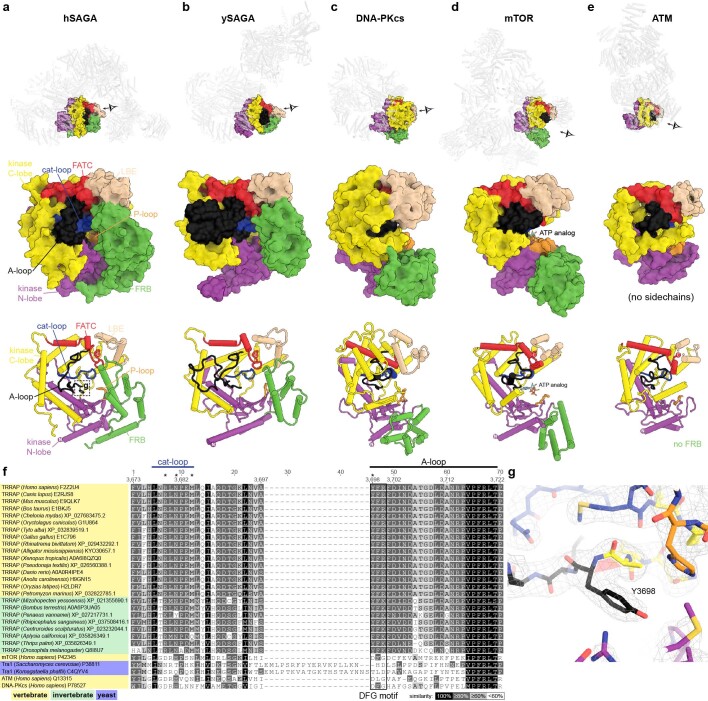
TRRAP ΨPIKK comparison and its integration into the HEAT repeat and FAT domain scaffold. **a-e**. ΨPIKK and its integration in hSAGA (a) and ySAGA^4^ (b, PDB: 6T9I), compared with the PIKK domains in DNA-PKc^70^ (c, PDB: 6ZFP), mTOR^71^ (d, PDB: 6BCX), and ATM^72^ (e, PDB: 5NP0). The panels below the overview show a close-up view as surface and cartoon representation of the active site entrance, as indicated in the top panels. Kinase elements are colored as indicated for hSAGA (FRB: FKBP-Rapamycin binding, P-loop: phosphate binding loop, LBE: mLST8 binding element, cat-loop: catalytic loop, FATC: FRAP-ATM-TRRAP C-terminus, A-loop: activation loop). For mTOR the kinase crystal structure with ATPγS^73^ (PDB: 4JSP) is shown. In agreement with the comparison by Díaz-Santín *et al*.^17^, the widely opened active site entrance in active kinases (**c-e**) is narrowed to a small tunnel in the pseudo-kinase in TRRAP (**a**) and Tra1 (**b**) by a rotation of the FRB domain. **f**. Sequence alignment of the catalytic and activation loop region of twenty-nine ΨPIKKs and PIKKs, colored by similarity as indicated. *: residues proposed to be involved in catalysis in mTOR^73^. **g**. Residue Y3698 of hSAGA, equivalent to the first residue in the DFG motif of the activation loop in active kinases^23^, adopts a cis-peptide bond, clearly defined by the density map (contoured at 9.0 σ).

TRRAP lacks kinase activity, although homologs of TRRAP are present in active kinases, such as mTOR, DNA-PKcs and ATM^28^ (Extended Data Fig. 9a–e). While the SAGA ΨPIKK lacks the canonical active site residues for catalysis^23,28^ (Extended Data Fig. 9f), we found that the first residue of the TRRAP activation loop (Y3698), corresponding to the aspartate in the active PIKK’s DFG motif^23^, adopts an unusual and well defined *cis*-peptide bond (Extended Data Fig. 9g). Such geometry outliers often serve a function in active sites^29^, and its position in our structure, together with the high evolutionary conservation of the ΨPIKK and of this specific residue in metazoans (Extended Data Fig. 9f), raises the question of whether the inactive kinase might have a different and so far undiscovered function, as observed for other pseudokinases^30^.

### Binding of InsP_6_ and its possible role in TRRAP stability

The resolution of our structure allowed us to visualize InsP_6_ in the positively charged pocket below the TRRAP FAT domain. The position of InsP_6_ in hSAGA is equivalent to that observed for mTORC2 (ref. ^31^) (Fig. 3b,e,f and Extended Data Fig. 6g,h) or the SMG1 kinase^32^, and thus it could serve a similar stabilizing role as proposed for those kinases^31,32^. In the ySAGA structures^4,5^, the region surrounding this pocket, including residues corresponding to R3051 and K3055 in hSAGA (Fig. 3f and Extended Data Fig. 6e), is poorly resolved and lacks InsP_6_ density (Extended Data Fig. 6i). On the other hand, an earlier structure of the yeast Tra1 subunit^17^ is better defined in this region and contains an unattributed density where InsP_6_ is seen bound in hSAGA (Extended Data Fig. 6j), potentially linking the stability of the TRRAP FAT domain to the presence of InsP_6_.

### Human disease mutations

**Fig. 5:**
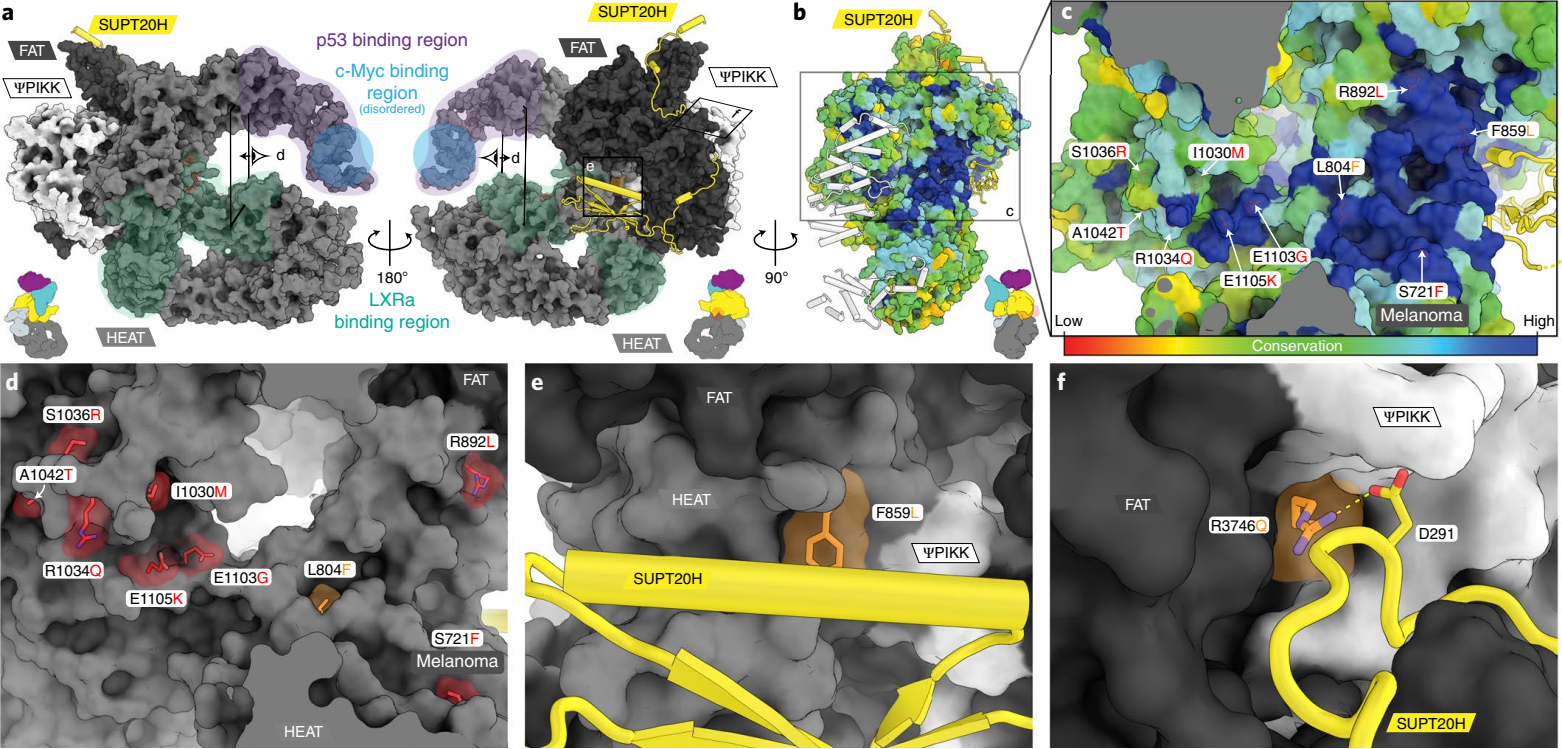
Activator binding and mapping of disease mutations in TRRAP. **a**, Surface representation of TRRAP colored by domains. Mapped activator-binding regions are indicated as colored overlays. The c-Myc binding region is probably located in a disordered loop between two helices. Boxes indicate the relative views in the following panels. **b**, Surface representation colored by conservation (for details, see Extended Data Fig. 6c). **c**,**d** Residue mutations associated with cancer, autism, or intellectual disability^34,37^. **c**, Most disease mutations lie in a region of high sequence conservation. Surface coloring as in **b**. **d**, Surface representation of disease mutations as shown in **a**. Red, surface exposed, probably interfere with activator binding; orange, buried, likely to structurally destabilize TRRAP (Extended Data Fig. 10a,b) or the interaction with SUPT20H (**e**,**f**). **e**, A disease-causing mutation of F859 is located at the interface with the SUPT20H CTD. **f**, R3746 forms a salt bridge with D291 of the SUPT20H latch. The disease mutation R3746Q disrupts the salt bridge. The reported residue numbers relate to the modeled isoform (Uniprot F2Z2U4).

**Extended Data Fig. 10:**
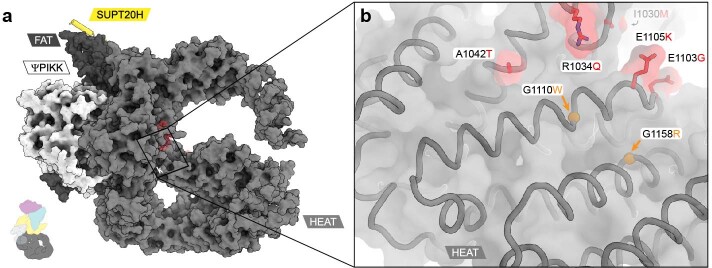
Buried mutations in the TRRAP module. **a**. Overview of TRRAP (as in Fig. 5a), with the rectangle indicating the location of mutations. **b**. Magnified view of the region showing how the mutations of the two buried glycine residues (G1110W and G1158R) in two neighboring helices are not compatible with bulky sidechain residues. A translucent surface representation is shown with a cartoon model of the TRRAP HEAT repeat domain. C∝ atoms are shown as spheres. Buried residues are shown in orange and surface accessible residues are shown in red (as in Fig. 5d).

The best characterized function of SAGA’s TRRAP module is serving as an interaction hub for transcriptional activators, which leads to its critical role in many diseases and its consideration as a prognostic marker and therapeutic target in many cancers^23,24,28,33–39^. Structurally, TRRAP displays high flexibility around the N-terminal cradle region where the c-Myc and p53 binding sites are located^24,36^ (Fig. 5a), and thus it is possible that c-Myc/p53 binding could stabilize or mediate conformational changes in this region. A cluster of disease-causing mutations lies along a highly conserved FAT-proximal HEAT repeat region where the N-terminal HEAT repeat arm and circular cradle meet (Fig. 5b,c), a site that has been shown to be crucial for liver X receptor alpha (LXRa) interaction^28,33,34,37^. A number of mutations, including the prevalent melanoma mutation S722F (TRRAP isoform here, S721F), are part of a highly conserved surface patch and probably involved in effector binding (Fig. 5c,d). Other mutations appear buried and are likely to affect folding of the HEAT repeats and interfere with the structural integrity of TRRAP (Extended Data Fig. 10a,b). Two independent mutations identified in patients with intellectual disability and neurodevelopmental disorders^37^ are at sites of interaction with the metazoan-specific extension seen in SUPT20H. The first (F859L) localizes directly at the interface with the SUPT20H CTD (Fig. 5e) and the second (R3746Q) eliminates a salt bridge with the highly conserved D291 of the SUPT20H latch (Fig. 5f and Extended Data Fig. 5b). Because TRRAP is a scaffold for other important cellular complexes, disease-causing mutations may also disrupt assembly or lead to perturbations within TIP60 (ref. ^28^).

## Concluding remarks

Our hSAGA structure reveals conserved structural elements as well as notable divergences from the yeast complex, including a distinct architecture and TRRAP–core interface, a lack of stable interaction with TBP and the visualization of the incorporation of the metazoan SPL module. Human SAGA complex combines transcription factor-interacting and enzymatic modules that need to regulate an intricate and unique transcriptional and chromatin landscape within human cells, in which enhancers and promoters are separated by kilo- to megabase distances. Furthermore, human promoter architectures, as well as intron and splice site properties, are very distinct from those in yeast^40,41^. These newly revealed structural features of hSAGA probably reflect unique mechanisms for this complex in human cells that go beyond transcription and chromatin regulation and can provide a launching point for further studies of SAGA’s roles in human disease.

## Methods

### SUPT7L-Halo-(FLAG)_3_ knock-in cell line generation

Human HeLa cells were cultured at 37 °C and 5% CO_2_ in 4.5 g l^−1^ glucose DMEM supplemented with 10% fetal bovine serum and 10 U ml^−1^ penicillin-streptomycin, and subcultured at a ratio of 1:3 to 1:8 every 2 to 4 d. Genome editing was performed as described previously^42^. Wild-type HeLa cells were cotransfected with a Cas9 plasmid (CBh-driven 3xFLAGSV40NLS-pSpCas9-NLS; PGK-driven mVenus; U6-driven single-guide (sg) RNA) and a repair plasmid containing Halo-(FLAG)_3_ flanked by roughly 800 bp of genomic homology sequence to *SUPT7L* on either side (18 μg of repair vector and 6 μg of Cas9 vector per P100 dish; 1:3 w/w) using Lipofectamine 2000 (Thermo Fisher catalog no. 11668019) according to the manufacturer’s protocol. Four sgRNAs were designed using the Zhang laboratory CRISPR design tool (https://zlab.bio/guide-design-resources), cloned into the Cas9 vector and cotransfected with the repair vector individually. After 18–24 h, transfected cells were combined and sorted using fluorescence activated cell sorting for mVenus fluorescence. mVenus-sorted cells were grown for 4–12 d, labeled with 500 nM Halo-TMR and cell populations with higher fluorescence than TMR-labeled wild-type cells were fluorescence activated cell sort-selected and sorted individually into 96-well plates. Clones were expanded and genotyped by PCR. Successfully edited clones were further verified by PCR using multiple primer combinations, Sanger sequencing and western blotting.

### Preparative HeLa cell culture and nuclei extraction

Large scale cultures of SUPT7L-Halo-(FLAG)_3_ HeLa cells were grown at 37 °C and ambient CO_2_ in a Hotpack Environmental Chamber (Scientific Products) in Joklik-modified Minimum Essential Medium Eagle (Sigma) media supplemented with 5% bovine calf serum, 50 U of penicillin-streptomycin and 2 mM Glutamax (Thermo Fisher). Cells were maintained in 6 l Florence round-bottom spinning flasks (Fisher Scientific) each containing 4 l of HeLa cultures and constantly stirred via a Precision Magnetic Stirrer Platform (Belloco). Every 24 h, cells were split 1:2 into fresh media grown to a density of roughly 2.5–5 × 10^5^ cells per ml and collected. To collect, SUPT7L-Halo-(FLAG)_3_ HeLa cells were centrifuged using a Fiberlite F9-6 ×1,000 LEX Fixed-Angle Rotor (Thermo Fisher) at 4 °C and 4,000 r.p.m. for 15 min. Cells were washed in PBSM (PBS buffer with 5 mM MgCl_2_) then centrifuged using an Eppendorf A-4-62 Swinging Bucket Rotor at 3,800 r.p.m. for 10 min. Cells were resuspended in 5 volumes of buffer A (10 mM HEPES pH 7.6, 1.5 mM MgCl_2_, 10 mM KCl, 1× Roche cOmplete protease inhibitors) briefly vortexed, incubated on ice for 20 min and centrifuged (Eppendorf A-4-62, 3,800 r.p.m., 10 min, 4 °C). Cells were lysed by resuspension in 2 volumes of buffer A and dounced seven times using a glass homogenizer with a type B pestle. Nuclei were pelleted by centrifugation (Eppendorf A-4-62, 2,700 r.p.m., 10 min, 4 °C), flash frozen in liquid nitrogen and stored at −80 °C until use.

### hSAGA purification

All steps were performed at 4 °C. Frozen nuclei from roughly 30 to 40 l of cell culture were thawed, 0.9 volumes of buffer C (20 mM HEPES pH 7.8, 1.5 mM MgCl_2_, 0.2 mM EDTA, 25% glycerol, 0.42 M KCl, 1 mM DTT, 0.5 mM phenylmethylsulfonyl fluoride (PMSF) and 1 μM Leupeptin) added and dounced using a glass homogenizer and a type B pestle 20 times on ice. The nuclear extract was then centrifuged using a JA-20 Beckman rotor at 4 °C and 20,000 r.p.m. for 30 min. The supernatant was collected and adjusted to a conductivity of 0.3 M NaCl. The nuclear extract (roughly 60 ml) was loaded onto a 50 ml phosphocellulose P11 (GE Healthcare/Whatman) column, washed with 3 column volumes (CVs) of 0.3 M NaCl HEMG (20 mM HEPES-KOH pH 7.6, 2 mM MgCl_2_, 0.2 mM EDTA, 10% glycerol, 1 mM DTT, 0.5 mM PMSF and 1 μM Leupeptin), then eluted in two steps with 3 CVs 0.5 M NaCl HEMG, followed by 3 CVs of 1.0 M NaCl HEMG and fractionated (5 ml). Peak fractions were determined by Bradford assay and combined. Human SAGA eluted with the 0.5 M NaCl HEMG peak (hereafter called P0.5 M) and dialyzed against 150 mM KCl buffer D (20 mM HEPES pH 7.8, 2 mM MgCl_2_, 0.2 mM EDTA, 10% glycerol, 150 mM KCl, 0.5 mM PMSF and 1 μM leupeptin) using SnakeSkin 10 kDa molecular weight cutoff dialysis tubing (Thermo Fisher). The dialyzed P0.5M fraction was supplemented with IGEPAL CA-630 (0.1% (v/v) final) and incubated with 500 μl of beads of FLAG M2 resin (Sigma) for 12 h nutating. The resin was washed twice with 2 CV of Column Buffer (25 mM HEPES pH 7.8, 0.2 M NaCl, 10% (v/v) glycerol, 1 mM EDTA, 0.5 mM TCEP, 0.1% (v/v) IGEPAL CA-630, 1× Roche cOmplete protease inhibitors), twice with 2 CV of Wash Buffer (Column buffer containing 0.6 M NaCl) and twice with 2 CV of Column Buffer. To elute, the beads were incubated with Column Buffer with 0.1 mg ml^−1^ 3xFLAG peptide rocked for 1 h, then centrifuged (Eppendorf 022653041 fixed-angle rotor, 3,300 r.p.m., 5 min) and this was repeated for four 1-h elutions. Elutions were concentrated fivefold using a 100 kDa molecular weight cutoff Spin-X UF concentrator (Corning). The sample was frozen in liquid nitrogen and stored at −80 °C. Sample quality and the effect of freeze–thaw cycles were analyzed by negative stain EM, and elution fractions yielded a similar quality in cryo-EM. A concentration of approximately 50 nM was determined by densitometry.

### Antibody information

The following primary antibodies were purchased from commercial suppliers and used at the indicated dilutions for western blotting. Anti-SUPT7L catalog no. sc-514548 (1:1,000) and anti-USP22 no. sc-390585 (1:200) were purchased from Santa Cruz Biotechnology. Anti-KAT2A catalog no. 3305 (1:1,000) was purchased from Cell Signaling Technology. Anti-TADA2B catalog no. PA5-57393 (1:2,500) was purchased from Thermo Fisher Scientific. Anti-TBP no. ab51841 (1:2,000) and anti-ENY2 no. ab183622 (1:1,000) were purchased from Abcam. Anti-TAF10 no. MABE1079 (1:2,000) was purchased from Millipore Sigma. Anti-TAF9B no. G2306 (1:500) is a homemade antibody previously created and validated in ref. ^43^.

### Mass spectrometry

Samples of purified hSAGA (roughly 1 µg) were shipped and analyzed by mass spectrometry by the Whitehead Institute Proteomics Facility (Cambridge, MA). Samples were diluted to 100 μl in 6 M urea, 100 mM Tris pH 7.8 buffer. Dithiothreitol (DTT, 5 μl of 200 mM) was added and incubated for 60 min at room temperature. Cysteines were alkylated by addition of 20 μl of 200 mM iodoacetamide and incubated for 60 min at room temperature. The sample was diluted to 900 μl with 100 mM Tris pH 7.8. The protein was digested by adding 100 μl of a 20 ng μl^−1^ trypsin or chymotrypsinin solution and incubated overnight at 37 °C. The resulting peptides were washed, extracted and concentrated by solid phase extraction using Waters Sep-Pak Plus C18 cartridges. Organic solvent was removed and volumes reduced to 15 μl by SpeedVac at 60 °C. The extracts were analyzed by reversed phase high-performance liquid chromatography using Waters NanoAcquity pumps and autosampler along with a Thermo Fisher Orbitrap Elite mass spectrometer using a nano flow configuration operated in a data dependent manner for 60 min. Fragmentation spectra were correlated against the Uniprot isoforms and TrEMBL databases for *Homo sapiens* using Sequest (Thermo Fisher Scientific; IseNode in Proteome Discoverer v.1.4.1.14). Sequest was searched (ion mass tolerance, 0.50 Da; parent ion tolerance, 15 ppm) with carbamidomethyl cysteine as fixed and methionine oxidation as variable modification. Consensus reports were obtained using Scaffold v.4.11.0 (Proteome Software Inc). Identified peptides were accepted with probabilities >95% (Scaffold local false discovery rate algorithm). Accepted protein identifications had a probability >99.0% and contained at least one identified peptide.

### Negative stain sample preparation of hSAGA, data collection and processing

Here, 400 mesh Cu grids were cleaned three times (in ethanol, water, ethanol) by sonication for 5 min and dried on filter paper. A petri dish was filled with water forming a meniscus and wiped off with lens paper. One drop of 1% (w/v) nitrocellulose in amyl acetate was added to the surface, forming a thin film. Cleaned grids were deposited on the film with the polished side facing down. The grids were transferred with parafilm onto filter paper with the nitrocellulose facing up and dried overnight. Grids were coated with carbon by evaporation using an Edwards Auto306 (10^−6^ mbar, 6 A, 6 s). Before sample adsorption, grids were glow discharged (30 s, 15 W, Tergo EM PIE scientific). Human SAGA was diluted (2×) in dilution buffer (25 mM HEPES pH 7.5, 0.2 mM EDTA, 6 mM MgCl_2_, 0.2 M NaCl, 3% (w/v) D(+) Trehalose), 3 µl were applied to a grid and adsorbed for 1 min. The grid was washed and stained, respectively, by swirling it five times on a 50 µl drop of 2% (w/v) uranyl formate for 10 s (each), blotted and dried in an air stream.

Data was collected on a Tecnai F20 (Thermo Fisher Scientific), using Leginon^44^ (Fig. 2 and Table 1). Micrographs were contrast transfer function (CTF) corrected using CTFfind v.4.1.13 (ref. ^45^). Particles were picked using Gaussian LoG picker in Relion-3.1 (ref. ^46^), extracted with a box size of 300 × 300 pixels and subjected to reference-free two-dimensional (2D) classification. Particles from the best classes (32%) were used for initial model generation using the statistical gradient descent method^47^ in Relion-3.1 (ref. ^46^). Particles were classified by a series of three-dimensional (3D) and 2D classifications with and without alignment (Extended Data Fig. 2a). Classification revealed one class without the (ordered) TAF6L HEAT domain and SPL module. Particles with and without this region were separated by multi-reference 3D classification. The best reconstruction was refined and classified again by alignment-free 3D classification. Combined classes that yielded the highest resolution were refined and then postprocessed in Relion v.3.1 (ref. ^46^) (Extended Data Fig. 2b).

### Cryo-EM sample preparation of hSAGA, data collection and processing

Quantifoil Au 300 mesh UltrAuFoil R1.2/1.3 polyethylenimine (PEI)/graphene-oxide grids were prepared according to established protocols^48^. The grids were used for freezing within 2–4 h.

All grid preparation steps were done on ice. Here, 3 µl of undiluted hSAGA was transferred into a 0.5 ml non-stick tube and crosslinked by mixing with 0.6 µl of crosslinking buffer (25 mM HEPES pH 7.8, 0.2 M NaCl, 0.2 mM EDTA, 0.5 mM TCEP, 0.01% (v/v) NP40, 10% (v/v) glycerol, 6 mM bis(sulfosuccinimidyl)suberate) and incubated for 5 min. A graphene-oxide grid was picked up with Vitrobot tweezers, the sample was transferred to the grid and incubated for 2 min in a saturated humidity chamber. Afterward, the grid was washed five times by submerging and swirling for 5 s (each) in 230 µl of wash buffer (25 mM HEPES pH 7.8, 0.2 M NaCl, 0.2 mM EDTA, 0.5 mM TCEP, 0.01% (v/v) NP40, 2.5% (v/v) glycerol) in a five-well Teflon block. Without letting the grid dry, excess solution was blotted off at a 90° angle and 4 µl of wash buffer were added immediately. The grid and tweezers were mounted into a Vitrobot Mark IV (Thermo Fisher Scientific), blotted with fresh filter paper (blot force 0, 3 s) and plunge frozen into liquid ethane.

Data were collected with SerialEM^49^ and 3 × 3 multishot acquisition on a Titan Krios G2 (Thermo Fisher Scientific) (Table 1). Videos were whole-frame motion corrected and binned (2×) using the Relion-3.1 (ref. ^46^), CTF corrected using CTFfind v.4.1.13 (ref. ^45^) and sorted manually. Particles were picked using the Gaussian LoG picker in Relion-3.1 (ref. ^46^) and extracted with 8× binning (Extended Data Fig. 3a) and a box size of 45 × 45 pixels. Graphene-oxide edges were removed by 2D classification before hSAGA particles could be classified. After removing most graphene-oxide edges, particles were reextracted with recentering (4× binned, 90 × 90 box size) and reclassified in 2D. The negative stain reconstruction was low-pass filtered to 50 Å and used as reference model for initial 3D classification. Each class was subclassified by alignment-free 2D classification to remove particles close or on graphene-oxide edges. The remaining particles were subjected to 3D classification, recentered in the box by applying a coordinate transformation to the particle alignment parameters using a custom python script, reextracted with recentering, without binning and placed in a box size of 360 × 360 pixels, then subjected to a consensus 3D refinement. Afterward, the particles were subjected to two rounds of Bayesian polishing, 3D refinement, CTF refinement and alignment-free 3D classification (tau = 20) (Extended Data Fig. 3a). A final round of 3D classification, refinement and postprocessing yielded a reconstruction at 2.93 Å (Extended Data Fig. 3b–d). High variability and low local resolution were observed at the TRRAP N terminus and the HEAT repeat cradle in close proximity as well as around the surface of the core module. Low-pass filtering and B factor blurring slightly improved the interpretability of the map in these regions. Further improvement was made by multibody refinement (Extended Data Figs. 3e and 8) of the core and TRRAP modules, although the resolution did not improve. Various density modification and map enhancement methods were tested, and the greatest improvements in variable and surface exposed regions were obtained by applying the spiral phase transform in LocSpiral^50^ to all reconstructions. This process revealed additional peptide connections and density fragments of the disordered TAF6L HEAT domain (Extended Data Fig. 3e,i,j). A principal component analysis of the multibody refinement showed a high degree of flexibility between the core and TRRAP modules (Extended Data Fig. 8a), which can also be observed by 3D variability analysis in Cryosparc v.2.15.0 (refs. ^47,51^). Signal subtraction after recentering and reextraction was attempted to detect density for the HAT and DUB modules but was not successful, presumably due to a high degree of conformational as well as potential compositional heterogeneity (metazoan SAGA has also been observed to occur without these modules^52,53^). Nevertheless, early samples that had been stabilized by GraFix^54^ revealed some 2D negative stain classes with highly variable density that is consistent with the suggested locations based on comparisons with ySAGA and the position of the N-terminal end of ATNX7 (Extended Data Fig. 4g). Masking and map transformations were carried out using UCSF Chimera^55^ and Relion-3.1 (ref. ^46^). All resolution estimates are based on the 0.143 threshold criterion of the gold-standard Fourier shell correlation (FSC)^56^ of two independently refined half sets in Relion-3.1 (ref. ^46^), after accounting for correlations introduced by masking^57^. Local resolution was estimated using Relion-3.1 (ref. ^46^).

### Cryo-EM sample preparation of hSAGA with TBP, data collection and processing

Human SAGA was mixed with a sixfold molar excess of human full length TBP and incubated for 5.2 h on ice. Grids were frozen and data were collected and processed in the same way as described above, but no additional density corresponding to TBP could be observed.

### Modeling and refinement

For model building in Coot^58^ maps were converted to structure factors using phenix.map_to_structure_factors^59^, allowing low-pass filtering and variable B-sharpening or blurring in Coot^58^. Models were built into the postprocessed, multibody-refined and LocSpiral^50^ filtered maps (Extended Data Fig. 3e–g). A fragmented initial model of secondary structure elements in TRRAP was generated using phenix.map_to_model^59^, manually corrected and completed in Coot^58^. A homology model of the TAF5L WD40 propeller was generated using SwissModel^60^ (based on Protein Data Bank (PDB) accession code 6F3T, ^61^) and rigid-body fitted in Coot^58^. The remaining model was built de novo in Coot^58^, guided by homology models based on human TFIID^9^ and ySAGA^4,5^. Regions with low confidence in register assignment were modeled as poly-alanines (assigned as unknown, UNKs). Before real-space refinement in Phenix, atomic B factors were reset to 90 Å^2^, the model was protonated using phenix.ready_set^59^ and sanity checked as well as geometry minimized using gelly^62^ (GlobalPhasing). Afterward, the model was refined using Rosetta^63^, validated using phenix.molprobity^59^ and optimized in Coot^58^. Secondary structure restraints were generated using phenix.secondary_structure_restraints and corrected after manual inspection. A final refinement was carried out with phenix.real_space_refine^59^ (1.18–3861) against the complete LocSpiral^50^ filtered map using default parameters plus secondary structure restraints, rotamers.fit=outliers_and_poormap and rotamers.tuneup=outliers_and_poormap (Extended Data Fig. 3g). Model statistics were calculated using phenix.molprobity^59^ (Table 1). Refinement against the regular postprocessed map resulted in almost identical statistics with an all-atom r.m.s.d. of 0.400 Å. All maps used for model building and refinement were deposited in the Electron Microscopy Data Bank (EMDB). Map versus model FSC was calculated using phenix.mtriage^59^ (Extended Data Fig. 3f). The InsP_6_ ligand was identified by density fit and homology to mTORC2 (ref. ^31^) and one out of two possible conformations was modeled (Fig. 3f and Extended Data Fig. 6e). In analysis of our cryo-EM structure, separating the core and TRRAP modules improved map quality and revealed additional features on surface exposed regions after LocSpiral filtering^50^ (Extended Data Fig. 3i,j). In particular, analysis of the region where SUPT3H, SUPT7L, TADA1 and TRRAP meet suggested alternative main chain conformations that could not be sorted out by classification. The highly variable region between SUPT3H, TADA1 and TRRAP corresponds to the approximate position where TBP binds to ySAGA (Extended Data Fig. 8b,c). The presence of multiple subunit isoforms, identified by mass spectrometry (Supplementary Table 2), did not affect modeling. Differences of isoforms are primarily located in disordered regions and were addressed according to PDB standards with remarks.

A model for the negative stain reconstruction was generated by rigid-body fitting without coordinate refinement in phenix.real_space_refine^59^ using the protein part of the cryo-EM model, a homology model of the TAF6L HEAT domain (generated with SwissModel^60^ and based on human TAF6, ref. ^9^, PDB 6MZL), and SF3B3/SF3B5 from the SF3b core complex^10^ (PDB 5IFE) (Table 1 and Extended Data Fig. 2d–i). Before fitting, expression tags in SF3B3 were deleted and the TAF6L HEAT domain was mutated to poly-alanines (annotated as UNKs) to reflect the absence of an authentic high- or medium-resolution structure for this region.

### Structural analysis and visualization

Coordinate transformations and manipulations were carried out using CCP4 tools^64^. Structures were compared using PDBefold^65^ and interfaces were analyzed using QtPISA v.2.1.0 (ref. ^64^). Relative angles between variable regions/domains (for example, TAF5(L) NTDs) of related structures with a common reference domain (for example, TAF5L WD40) were calculated by prealigning all structures to the reference domain of hSAGA using secondary structure matching. The center of masses of the hSAGA reference domain (for example, TAF5L WD40), the hSAGA variable domain (for example, TAF5L NTD or TRRAP ΨPIKK) and the hSAGA variable domain after superposition on the corresponding domain in related structures using secondary structure matching (for example, ySAGA Taf5 NTD or Tra1 ΨPIKK) were calculated. Center of masses were calculated in PyMOL (The PyMOL Molecular Graphics System, v.2.4.0 Schrödinger, LLC.) and angles between corresponding vectors were calculated using python. Related structures were identified using PDBeFold (70% query/70% target)^65^. Structure figures were generated using PyMOL, ChimeraX (UCSF, 2020-01-10) and Adobe Illustrator. Electrostatic surfaces were generated using the APBS^66^ plugin in PyMOL. Videos were generated using ChimeraX^67^ (UCSF, 2020-01-10), Adobe Premier and ffmpeg (https://ffmpeg.org). Plots were generated using python. Reported contour levels for maps are defined as *σ* = density threshold/r.m.s.

### Sequence analysis

In total, 23 metazoan homologs of hSAGA with a complete set of all 20 subunits were retrieved from databases. Sequence alignments were generated using the Clustal Omega^68^ executable in Geneious Prime v.2021.0.3. Sequence conservation figures were generated by aligning all subunit sequences of all 23 metazoan SAGAs with the sequences of the molecular model of hSAGA. Alignments were combined and conservation scores were calculated using AL2CO^69^ and used for coloring in PyMOL.

### Reporting Summary

Further information on research design is available in the Nature Research Reporting Summary linked to this article.

## Online content

Any methods, additional references, Nature Research reporting summaries, source data, extended data, supplementary information, acknowledgements, peer review information; details of author contributions and competing interests; and statements of data and code availability are available at 10.1038/s41594-021-00682-7.

## Supplementary information

Supplementary Information Supplementary Tables 1–3 and Fig. 1.

Reporting Summary

Supplementary Video 1 Overview of the negative stain and cryo-EM reconstructions and the atomic model of hSAGA. The .mp4 video displays the 3D negative stain reconstruction (19 Å) followed by the cryo-EM reconstruction (2.9 Å) of hSAGA. The two reconstructions are then overlayed to show the incorporation of the TAF6L HEAT domain and the SPL module. The subunits are labeled and colored as in Fig. 1. The end shows the atomic model of hSAGA.

## Data Availability

Cryo-EM maps and refined coordinates were deposited in the EMDB with accession codes EMD-23027 and EMD-23028 and in the PDB with accession codes 7KTR and 7KTS. The cell line can be provided on request. Supplementary Information is linked to the online version of the paper at www.nature.com/nature. Correspondence should be addressed to enogales@lbl.gov. Source data are provided with this paper.

## Source data

Source Data Extended Data Fig. 1 Uncropped scan with size marker indication (from Extended Data Fig. 1b) and uncropped western blot imaged with chemiluminescence (from Extended Data Fig. 1c).

## Extended data

are available for this paper at 10.1038/s41594-021-00682-7.

## Code availability

Custom computer code is available on Github (coord_transform_to_star, https://github.com/dominikaherbst/cryo-em_scripts).

## References

[1] Timmers, H. T. M. SAGA and TFIID: friends of TBP drifting apart. *Biochim. Biophys. Acta Gene Regul. Mech*. **1864**, 10.1016/j.bbagrm.2020.194604 (2021). 10.1016/j.bbagrm.2020.194604 32673655

[2] Fischer V, Schumacher K, Tora L, Devys D (2019). Global role for coactivator complexes in RNA polymerase II transcription. Transcription.

[3] Wang L, Dent SY (2014). Functions of SAGA in development and disease. Epigenomics.

[4] Wang H (2020). Structure of the transcription coactivator SAGA. Nature.

[5] Papai G (2020). Structure of SAGA and mechanism of TBP deposition on gene promoters. Nature.

[6] Helmlinger D, Tora L (2017). Sharing the SAGA. Trends Biochem. Sci..

[7] Cheon Y, Kim H, Park K, Kim M, Lee D (2020). Dynamic modules of the coactivator SAGA in eukaryotic transcription. Exp. Mol. Med..

[8] Antonova, S. V., Boeren, J., Timmers, H. T. M. & Snel, B. Epigenetics and transcription regulation during eukaryotic diversification: the saga of TFIID. *Gene Dev.* **33**, 10.1101/gad.300475.117 (2019). 10.1101/gad.300475.117 PMC6672047 31123066

[9] Patel, A. B. et al. Structure of human TFIID and mechanism of TBP loading onto promoter DNA. *Science* **362**, 10.1126/science.aau8872 (2018). 10.1126/science.aau8872 PMC6446905 30442764

[10] Cretu C (2016). Molecular architecture of SF3b and structural consequences of its cancer-related mutations. Mol. Cell.

[11] Kolesnikova O (2018). Molecular structure of promoter-bound yeast TFIID. Nat. Commun..

[12] Chen, X. et al. Structural insights into preinitiation complex assembly on core promoters. *Science* **372**, 10.1126/science.aba8490 (2021). 10.1126/science.aba8490 33795473

[13] Stegeman R (2016). The spliceosomal protein SF3B5 is a novel component of *Drosophila* SAGA that functions in gene expression independent of splicing. J. Mol. Biol..

[14] Elias-Villalobos A, Toullec D, Faux C, Seveno M, Helmlinger D (2019). Chaperone-mediated ordered assembly of the SAGA and NuA4 transcription co-activator complexes in yeast. Nat. Commun..

[15] Nagy Z (2009). The Human SPT20-containing SAGA complex plays a direct role in the regulation of endoplasmic reticulum stress-induced genes. Mol. Cell. Biol..

[16] Dengl S, Mayer A, Sun M, Cramer P (2009). Structure and in vivo requirement of the yeast Spt6 SH2 domain. J. Mol. Biol..

[17] Diaz-Santin, L. M., Lukoyanova, N., Aciyan, E. & Cheung, A. C. Cryo-EM structure of the SAGA and NuA4 coactivator subunit Tra1 at 3.7 angstrom resolution. *eLife* **6**, 10.7554/eLife.28384 (2017). 10.7554/eLife.28384 PMC5576489 28767037

[18] Sharov G (2017). Structure of the transcription activator target Tra1 within the chromatin modifying complex SAGA. Nat. Commun..

[19] Setiaputra D (2015). Conformational flexibility and subunit arrangement of the modular yeast Spt-Ada-Gcn5 acetyltransferase complex. J. Biol. Chem..

[20] Wang X, Ahmad S, Zhang Z, Cote J, Cai G (2018). Architecture of the *Saccharomyces cerevisiae* NuA4/TIP60 complex. Nat. Commun..

[21] Sermwittayawong D, Tan S (2006). SAGA binds TBP via its Spt8 subunit in competition with DNA: implications for TBP recruitment. EMBO J..

[22] Wei Y (2019). Multiple direct interactions of TBP with the MYC oncoprotein. Nat. Struct. Mol. Biol..

[23] McMahon SB, Van Buskirk HA, Dugan KA, Copeland TD, Cole MD (1998). The novel ATM-related protein TRRAP is an essential cofactor for the c-Myc and E2F oncoproteins. Cell.

[24] Feris EJ, Hinds JW, Cole MD (2019). Formation of a structurally-stable conformation by the intrinsically disordered MYC:TRRAP complex. PLoS ONE.

[25] Brand M (2001). UV-damaged DNA-binding protein in the TFTC complex links DNA damage recognition to nucleosome acetylation. EMBO J..

[26] Martinez E (2002). Human STAGA complex is a chromatin-acetylating transcription coactivator that interacts with pre-mRNA splicing and DNA damage-binding factors in vivo. Mol. Cell. Biol..

[27] Sun C (2020). The SF3b complex: splicing and beyond. Cell Mol. Life Sci..

[28] Murr R, Vaissiere T, Sawan C, Shukla V, Herceg Z (2007). Orchestration of chromatin-based processes: mind the TRRAP. Oncogene.

[29] Weiss MS, Jabs A, Hilgenfeld R (1998). Peptide bonds revisited. Nat. Struct. Biol..

[30] Reiterer V, Eyers PA, Farhan H (2014). Day of the dead: pseudokinases and pseudophosphatases in physiology and disease. Trends Cell Biol..

[31] Scaiola, A. et al. The 3.2-A resolution structure of human mTORC2. *Sci. Adv.* **6**, 10.1126/sciadv.abc1251 (2020). 10.1126/sciadv.abc1251 PMC7673708 33158864

[32] Gat Y (2019). InsP6 binding to PIKK kinases revealed by the cryo-EM structure of an SMG1-SMG8-SMG9 complex. Nat. Struct. Mol. Biol..

[33] Unno A (2005). TRRAP as a hepatic coactivator of LXR and FXR function. Biochem. Bioph. Res. Co..

[34] Wei X (2011). Exome sequencing identifies GRIN2A as frequently mutated in melanoma. Nat. Genet..

[35] Wang J (2016). Analysis of TRRAP as a potential molecular marker and therapeutic target for breast cancer. J. Breast Cancer.

[36] Ard PG (2002). Transcriptional regulation of the mdm2 oncogene by p53 requires TRRAP acetyltransferase complexes. Mol. Cell Biol..

[37] Cogne B (2019). Missense variants in the histone acetyltransferase complex component gene TRRAP cause autism and syndromic intellectual disability. Am. J. Hum. Genet..

[38] McMahon SB, Wood MA, Cole MD (2000). The essential cofactor TRRAP recruits the histone acetyltransferase hGCN5 to c-Myc. Mol. Cell Biol..

[39] Herceg Z (2001). Disruption of Trrap causes early embryonic lethality and defects in cell cycle progression. Nat. Genet..

[40] Dobi KC, Winston F (2007). Analysis of transcriptional activation at a distance in *Saccharomyces cerevisiae*. Mol. Cell Biol..

[41] Lenhard B, Sandelin A, Carninci P (2012). Metazoan promoters: emerging characteristics and insights into transcriptional regulation. Nat. Rev. Genet..

[42] Hansen, A. S., Pustova, I., Cattoglio, C., Tjian, R. & Darzacq, X. CTCF and cohesin regulate chromatin loop stability with distinct dynamics. *eLife* **6**, 10.7554/eLife.25776 (2017). 10.7554/eLife.25776 PMC5446243 28467304

[43] Herrera FJ, Yamaguchi T, Roelink H, Tjian R (2014). Core promoter factor TAF9B regulates neuronal gene expression. eLife.

[44] Suloway C (2005). Automated molecular microscopy: the new Leginon system. J. Struct. Biol..

[45] Rohou A, Grigorieff N (2015). CTFFIND4: fast and accurate defocus estimation from electron micrographs. J. Struct. Biol..

[46] Zivanov, J. et al. New tools for automated high-resolution cryo-EM structure determination in RELION-3. *eLife* **7**, 10.7554/eLife.42166 (2018). 10.7554/eLife.42166 PMC6250425 30412051

[47] Punjani A, Rubinstein JL, Fleet DJ, Brubaker MA (2017). cryoSPARC: algorithms for rapid unsupervised cryo-EM structure determination. Nat. Methods.

[48] Patel, A., Toso, D., Litvak, A. & Nogales, E. Efficient graphene oxide coating improves cryo-EM sample preparation and data collection from tilted grids. Preprint at *bioRxiv*10.1101/2021.03.08.434344 (2021).

[49] Schorb M, Haberbosch I, Hagen WJH, Schwab Y, Mastronarde DN (2019). Software tools for automated transmission electron microscopy. Nat. Methods.

[50] Kaur S (2021). Local computational methods to improve the interpretability and analysis of cryo-EM maps. Nat. Commun..

[51] Punjani A, Fleet DJ (2021). 3D variability analysis: resolving continuous flexibility and discrete heterogeneity from single particle cryo-EM. J. Struct. Biol..

[52] Soffers JHM (2019). Characterization of a metazoan ADA acetyltransferase complex. Nucleic Acids Res..

[53] Li X (2017). Enzymatic modules of the SAGA chromatin-modifying complex play distinct roles in *Drosophila* gene expression and development. Genes Dev..

[54] Kastner B (2008). GraFix: sample preparation for single-particle electron cryomicroscopy. Nat. Methods.

[55] Pettersen EF (2004). UCSF Chimera-a visualization system for exploratory research and analysis. J. Comput. Chem..

[56] Rosenthal PB, Henderson R (2003). Optimal determination of particle orientation, absolute hand, and contrast loss in single-particle electron cryomicroscopy. J. Mol. Biol..

[57] Chen S (2013). High-resolution noise substitution to measure overfitting and validate resolution in 3D structure determination by single particle electron cryomicroscopy. Ultramicroscopy.

[58] Emsley P, Cowtan K (2004). Coot: model-building tools for molecular graphics. Acta Crystallogr. Sect. D., Biol. Crystallogr..

[59] Adams PD (2010). PHENIX: a comprehensive Python-based system for macromolecular structure solution. Acta Crystallogr. D. Biol. Crystallogr..

[60] Schwede T, Kopp J, Guex N, Peitsch MC (2003). SWISS-MODEL: an automated protein homology-modeling server. Nucleic Acids Res..

[61] Antonova SV (2018). Chaperonin CCT checkpoint function in basal transcription factor TFIID assembly. Nat. Struct. Mol. Biol..

[62] Bricogne, G. et al. BUSTER v.2.10.3 (Global Phasing Ltd, 2011).

[63] DiMaio F, Tyka MD, Baker ML, Chiu W, Baker D (2009). Refinement of protein structures into low-resolution density maps using rosetta. J. Mol. Biol..

[64] Collaborative Computational Project, N. (1994). The CCP4 suite: programs for protein crystallography. Acta Crystallogr. Sect. D..

[65] Krissinel E, Henrick K (2004). Secondary-structure matching (SSM), a new tool for fast protein structure alignment in three dimensions. Acta Crystallogr. D. Biol. Crystallogr..

[66] Baker NA, Sept D, Joseph S, Holst MJ, McCammon JA (2001). Electrostatics of nanosystems: application to microtubules and the ribosome. Proc. Natl Acad. Sci. USA.

[67] Pettersen EF (2020). UCSF ChimeraX: Structure visualization for researchers, educators, and developers. Protein Sci..

[68] Sievers F (2011). Fast, scalable generation of high-quality protein multiple sequence alignments using Clustal Omega. Mol. Syst. Biol..

[69] Pei J, Grishin NV (2001). AL2CO: calculation of positional conservation in a protein sequence alignment. Bioinformatics.

