# Supplementary material for: Structure of the human SAGA coactivator complex
Source: Nat Struct Mol Biol. 2021 Nov 22;28(12):989–96. doi: 10.1038/s41594-021-00682-7 (PMC8660637; doi:10.1038/s41594-021-00682-7)
Supplement: Supplementary file 1 — Supplementary Tables 1–3 and Fig. 1. [file 41594_2021_682_MOESM1_ESM.pdf]

nature portfolio
https://doi.org/10.1038/s41594-021-00682-7

# Supplementary information

# Structure of the human SAGA coactivator complex

In the format provided by the authors and unedited

**Supplementary Information for**

# Structure of the human SAGA coactivator complex

Dominik A. Herbst[1,2,&], Meagan N. Esbin[3,4,&], Robert K. Louder[1,4,†], Claire Dugast-Darzacq[3], Gina M. Dailey[3], Qianglin Fang[1,2,§], Xavier Darzacq[3], Robert Tjian[3,5], Eva Nogales[1,2,3,5,*]

[1] California Institute for Quantitative Biology (QB3), University of California, Berkeley, CA 94720.

[2] Molecular Biophysics and Integrated Bio-Imaging Division, Lawrence Berkeley National Laboratory, Berkeley, CA 94720, USA.

[3] Department of Molecular and Cell Biology, University of California, Berkeley, CA 94720, USA.

[4] Biophysics Graduate Group, University of California, Berkeley, CA 94720, USA.

[5] Howard Hughes Medical Institute, University of California, Berkeley, CA 94720, USA.

[†] Current address: Department of Biology, Johns Hopkins University, Baltimore, Maryland 21218, USA.

[§] Current address: School of Public Health, Sun Yat-sen University, Shenzhen, 510006 Guangzhou, China

[&] These authors contributed equally to this work

[*] Corresponding author. Email: enogales@lbl.gov

**Supplementary Table 1 | Sequence conservation of SAGA subunits in metazoan.**

| Common name | Scientific name | ATXN7 | ATXN7L3 | ENY2 | USP22 | KAT2A | SGF29 | TADA2B | TADA3 | SF3B3 | SF3B5 | SUPT20H | SUPT3H | SUPT7L | TADA1 | TAF10 | TAF12 | TAF5L | TAF6L | TAF9B | TRRAP | average |
|---|---|---|---|---|---|---|---|---|---|---|---|---|---|---|---|---|---|---|---|---|---|---|
| Human[m,v] | *Homo sapiens* | 100 | 100 | 100 | 100 | 100 | 100 | 100 | 100 | 100 | 100 | 100 | 100 | 100 | 100 | 100 | 100 | 100 | 100 | 100 | 100 | **100** |
| Mouse[m,v] | *Mus musculus* | 84 | 99 | 100 | 98 | 97 | 98 | 99 | 99 | 100 | 99 | 91 | 78 | 95 | 96 | 92 | 96 | 93 | 93 | 90 | 99 | **95** |
| Cattle[m,v] | *Bos taurus* | 89 | 99 | 100 | 97 | 98 | 99 | 83 | 100 | 100 | 100 | 86 | 80 | 97 | 97 | 93 | 99 | 94 | 94 | 85 | 99 | **94** |
| Dog[m,v] | *Canis lupus* | 93 | 97 | 95 | 98 | 98 | 95 | 23 | 100 | 100 | 100 | 82 | 68 | 98 | 99 | 97 | 99 | 99 | 94 | 94 | 99 | **91** |
| Rabbit[m,v] | *Oryctolagus cuniculus* | 91 | 97 | 78 | 97 | 94 | 91 | 23 | 99 | 97 | 100 | 82 | 91 | 96 | 98 | 95 | 99 | 99 | 89 | 95 | 97 | **90** |
| Alligator[v] | *Alligator mississippiensis* | 73 | 86 | 99 | 94 | 84 | 96 | 96 | 77 | 99 | 98 | 81 | 63 | 87 | 89 | 93 | 94 | 92 | 71 | 77 | 95 | **87** |
| Barn owl[v] | *Tyto alba* | 72 | 68 | 98 | 96 | 89 | 74 | 97 | 92 | 98 | 98 | 80 | 70 | 87 | 90 | 49 | 94 | 93 | 88 | 74 | 97 | **85** |
| Sea turtle[v] | *Chelonia mydas* | 36 | 86 | 98 | 94 | 87 | 97 | 96 | 92 | 99 | 99 | 80 | 67 | 88 | 88 | 64 | 93 | 93 | 71 | 79 | 97 | **85** |
| Snake[v] | *Pseudonaja textilis* | 66 | 86 | 98 | 91 | 82 | 97 | 93 | 89 | 99 | 97 | 73 | 67 | 85 | 89 | 81 | 90 | 90 | 64 | 75 | 93 | **85** |
| Lizard[v] | *Anolis carolinensis* | 72 | 81 | 98 | 89 | 82 | 96 | 94 | 92 | 99 | 97 | 67 | 66 | 86 | 84 | 51 | 92 | 92 | 68 | 76 | 90 | **84** |
| Two-lined caecilian[v] | *Rhinatrema bivittatum* | 63 | 84 | 99 | 94 | 69 | 96 | 90 | 92 | 97 | 97 | 72 | 66 | 79 | 70 | 63 | 91 | 86 | 68 | 78 | 96 | **83** |
| Chicken[v] | *Gallus gallus* | 69 | 86 | 96 | 92 | 84 | 95 | 21 | 93 | 99 | 98 | 71 | 67 | 87 | 84 | 67 | 95 | 92 | 59 | 79 | 97 | **82** |
| Japanese rice fish[v] | *Oryzias latipes* | 41 | 65 | 81 | 91 | 81 | 90 | 85 | 79 | 93 | 94 | 60 | 49 | 64 | 60 | 60 | 84 | 68 | 61 | 70 | 89 | **73** |
| Zebrafish[v] | *Danio rerio* | 42 | 68 | 85 | 92 | 83 | 91 | 25 | 78 | 93 | 94 | 62 | 57 | 70 | 65 | 61 | 79 | 67 | 63 | 74 | 92 | **72** |
| Frog[v] | *Xenopus tropicalis* | 4 | 11 | 94 | 92 | 7 | 96 | 8 | 88 | 97 | 95 | 70 | 66 | 79 | 81 | 64 | 76 | 81 | 58 | 77 | 94 | **67** |
| Lamprey[v] | *Petromyzon marinus* | 21 | 50 | 80 | 82 | 70 | 85 | 31 | 66 | 92 | 90 | 41 | 39 | 45 | 44 | 61 | 50 | 34 | 46 | 58 | 78 | **58** |
| Scallop[i] | *Mizuhopecten yessoensis* | 13 | 38 | 83 | 60 | 58 | 68 | 38 | 45 | 83 | 81 | 24 | 35 | 29 | 38 | 57 | 59 | 39 | 32 | 38 | 62 | **49** |
| Scorpion[i] | *Centruroides sculpturatus* | 14 | 39 | 73 | 57 | 61 | 65 | 41 | 46 | 84 | 76 | 21 | 20 | 30 | 40 | 58 | 49 | 31 | 30 | 46 | 56 | **47** |
| Tick[i] | *Rhipicephalus sanguineus* | 14 | 39 | 63 | 58 | 58 | 63 | 44 | 34 | 81 | 81 | 20 | 27 | 23 | 35 | 52 | 41 | 39 | 28 | 38 | 56 | **45** |
| Sea hare[i] | *Aplysia californica* | 13 | 32 | 79 | 57 | 54 | 56 | 37 | 40 | 81 | 78 | 23 | 32 | 24 | 33 | 58 | 38 | 35 | 23 | 41 | 56 | **45** |
| Bumblebee[i] | *Bombus terrestris* | 12 | 30 | 51 | 59 | 51 | 55 | 34 | 32 | 80 | 86 | 16 | 36 | 18 | 28 | 57 | 43 | 29 | 15 | 31 | 58 | **41** |
| Thrip[i] | *Thrips palmi* | 12 | 29 | 52 | 54 | 49 | 54 | 37 | 27 | 81 | 85 | 14 | 26 | 18 | 34 | 59 | 36 | 28 | 18 | 29 | 54 | **40** |
| Shrimp[i] | *Penaeus vannamei* | 12 | 30 | 59 | 18 | 54 | 48 | 43 | 37 | 79 | 76 | 23 | 32 | 24 | 28 | 55 | 33 | 29 | 16 | 34 | 57 | **39** |
| Fruit fly[i] | *Drosophila melanogaster* | 9 | 30 | 44 | 50 | 43 | 47 | 21 | 24 | 75 | 84 | 14 | 20 | 14 | 24 | 36 | 37 | 26 | 17 | 27 | 50 | **35** |
| Pichia pastoris[y] | *Komagataella phaffii* | 8 | 18 | 25 | 29 | 34 | 13 | 23 | 12 | N/A | N/A | 8 | 20 | 11 | 15 | 24 | 21 | 25 | 16 | 31 | 25 | **18** |
| Brewer's yeast[y] | *Saccharomyces cerevisiae* | 8 | 23 | 27 | 25 | 34 | 15 | 24 | 13 | N/A | N/A | 7 | 23 | 10 | 14 | 21 | 18 | 24 | 15 | 31 | 25 | **18** |

Sequence identities (%) and subunit names correspond to the human homologs and are sorted by decreasing average identity. N/A: not applicable (no known homolog). Vertebrates have an average sequence identity of ≥ 60%. Indices indicate classification into mammals (m), vertebrate (v), invertebrate (i), and yeast (y).

**Supplementary Table 2 | Mass spectrometric identification of the purified hSAGA sample.**

| Total Spectrum Count | Accession Number | Alternative Isoforms | SAGA Subunit Name | Modeled | SAGA Module | Molecular Weight | # Amino Acids | PSM/AA | Norm. Prop. to TRRAP |
|---|---|---|---|---|---|---|---|---|---|
| 771 | Q9Y4A5 | Q9Y4A5-2 | TRRAP | | TRRAP | 438 kDa | 3859 | 0.200 | 1.01 |
| 762 | F2Z2U4 | | TRRAP | Y | TRRAP | 436 kDa | 3848 | 0.198 | 1.00 |
| 176 | Q92830 | | KAT2A | | HAT | 94 kDa | 837 | 0.210 | 1.06 |
| 171 | O75529 | | TAF5L | Y | Core | 66 kDa | 589 | 0.290 | 1.47 |
| 151 | Q15393 | | SF3B3 | Y | SPL | 136 kDa | 1217 | 0.124 | 0.63 |
| 134 | Q8NEM7-3 | Q8NEM7-2, R4GND2 | SUPT20H | Y | Core | 88 kDa | 811 | 0.165 | 0.83 |
| 122 | Q9Y6J9 | | TAF6L | Y | Core | 68 kDa | 622 | 0.196 | 0.99 |
| 111 | Q86TJ2 | | TADA2b | | HAT | 48 kDa | 420 | 0.264 | 1.33 |
| 108 | O75528 | | TADA3 | | HAT | 49 kDa | 432 | 0.250 | 1.26 |
| 106 | O15265 | | ATXN7 | Y | DUB | 95 kDa | 892 | 0.119 | 0.60 |
| 90 | Q96BN2 | | TADA1 | Y | Core | 37 kDa | 335 | 0.269 | 1.36 |
| 78 | Q9UPT9 | | USP22 | | DUB | 60 kDa | 525 | 0.149 | 0.75 |
| 65 | Q92831 | | KAT2B | | HAT | 93 kDa | 832 | 0.078 | 0.39 |
| 63 | Q9HBM6 | | TAF9B | Y | Core | 28 kDa | 251 | 0.251 | 1.27 |
| 62 | Q96ES7 | | SGF29 | | HAT | 33 kDa | 293 | 0.212 | 1.07 |
| 57 | Q9ULK2 | A4D0Q3 | ATXN7L1 | | DUB | 92 kDa | 861 | 0.066 | 0.33 |
| 53 | O94864 | O94864-2 | SUPT7L | Y | Core | 46 kDa | 414 | 0.128 | 0.65 |
| 51 | Q5T6C5 | | ATXN7L2 | | DUB | 77 kDa | 722 | 0.071 | 0.36 |
| 49 | A0A3B3ITZ9 | Q9Y2W1 | | | | | | | |
| 47 | Q14CW9 | Q14CW9-2 | ATXN7L3 | | DUB | 39 kDa | 347 | 0.135 | 0.68 |
| 43 | Q16594 | | TAF9 | | Core | 29 kDa | 264 | 0.163 | 0.82 |
| 40 | Q12962 | | TAF10 | Y | Core | 22 kDa | 218 | 0.183 | 0.93 |
| 36 | O75486 | O75486-4, Q5U608 | SUPT3H | Y | Core | 36 kDa | 317 | 0.114 | 0.57 |
| 29 | A0A4D5RAC7 | A0A4D5RAC9, P68104, Q53G85, Q53GE9, Q53HQ7, Q53HR5, Q6IPN6, QPIPT9 | | | | | | | |
| 27 | A0A1W2PPS1 | B4DLR3, Q00839, Q00839-2 | | | | | | | |
| 25 | Q9BWJ5 | | SF3B5 | Y | SPL | 10 kDa | 86 | 0.291 | 1.47 |
| 22 | Q1KMD3 | | | | | | | | |
| 21 | Q16514 | Q16514-2 | TAF12 | Y | Core | 18 kDa | 161 | 0.130 | 0.66 |
| 19 | P60709 | P63261, Q53G76, Q53GK6 | | | | | | | |
| 17 | Q9NPA8 | Q9NPA8-2 | ENY2 | | DUB | 12 kDa | 101 | 0.168 | 0.85 |

| | | |
|---|---|---|
| 17 | P84090 | |
| 13 | P11142 | P11142-2, Q53HF2 |
| 12 | E9PK91 | Q9NYF8, Q9NYF8-2, Q9NYF8-3 |
| 10 | Q8NF21 | |
| 10 | B4DE59 | |
| 5 | Q14966 | Q14966-3 |
| 5 | Q9Y3Y2 | Q9Y3Y2-3, Q9Y3Y2-4, X6R700 |
| 5 | A0A0G2JIW1 | A0A1U9X7W4, B4DFN9, P0DMV8, P0DMV8-2, Q59EJ3 |
| 5 | B2RBD5 | |
| 4 | Q6UWP8 | |
| 4 | I0B0K3 | I0B0K4, I0B0K5, I0B0K6, I0B0K7, I0B0K8, P20930, Q05331 |
| 4 | P67809 | |
| 4 | P31151 | |
| 3 | M0R0R2 | P46782 |
| 3 | P06702 | |
| 3 | P04406 | P04406-2, Q0QET7, Q2TSD0 |
| 3 | Q53GA7 | |
| 3 | A6NMY6 | |
| 2 | P11021 | |
| 2 | C9J352 | C9J4U1, C9JWD9, C9JYI4, F2Z3G4, P52294, Q5BKZ2 |
| 2 | Q05DU1 | Q05DU1, Q9NVN8 |

Proteins are shown as identified only if the protein probability is >99% and the peptide threshold is >95%. Isoforms indistinguishable by MS are listed in Column 3. If multiple isoforms were indistinguishable by MS, the modeled or canonical isoform is listed in Column 2. For hSAGA subunits only, more detailed information is shown in Columns 4-10. A "Y" in the Modeled column indicates that subunit or isoform was modeled in the hSAGA structure. *PSM/AA equals Total Spectrum Count divided by # Amino Acids. Column 10 shows PSM/AA values normalized to TRRAP=1 to show the approximate stoichiometry of each subunit.

**Supplementary Table 3 | Core – TRRAP/Tra1 interface areas**

| TRRAP / Tra1 vs | All | All except SUPT20H/Spt20 | All except TAF12/Taf12 | All except SUPT20H/Spt20 TAF12/Taf12 | SUPT20H/Spt20 | TAF12 / Taf12 |
|---|---|---|---|---|---|---|
| **Complete interface** | | | | | | |
| human, PDB: 7KTR | 7,073 | 2,661 | 6,927 | 2,514 | 4,453 | 179 |
| yeast (*S. cerevisiae*), PDB: 6T9I | 4,427 | 2,880 | 1,726 | 0 | 1,708 | 2,862 |
| yeast (*K. phaffii*), PDB: 6TB4 | 4,217 | 2,930 | 1,960 | 582 | 1,370 | 2,339 |
| **In core interface** | | | | | | |
| human, PDB: 7KTR | 3,545 | 2,661 | 3,400 | 2,514 | 925 | 179 |
| yeast (*S. cerevisiae*), PDB: 6T9I | 3,291 | 1,744 | 1,726 | 0 | 1,708 | 1,726 |
| yeast (*K. phaffii*), PDB: 6TB4 | 3,532 | 2,244 | 1,960 | 582 | 1,370 | 1,654 |
| **In extended interface** | | | | | | |
| human, PDB: 7KTR | 3,528 | 0 | 3,528 | 0 | 3,528 | 0 |
| yeast (*S. cerevisiae*), PDB: 6T9I | 1,152 | 1,152 | 0 | 0 | 0 | 1,152 |
| yeast (*K. phaffii*), PDB: 6TB4 | 686 | 686 | 0 | 0 | 0 | 686 |

Table with interface areas corresponding to Extended Data Fig. 7.

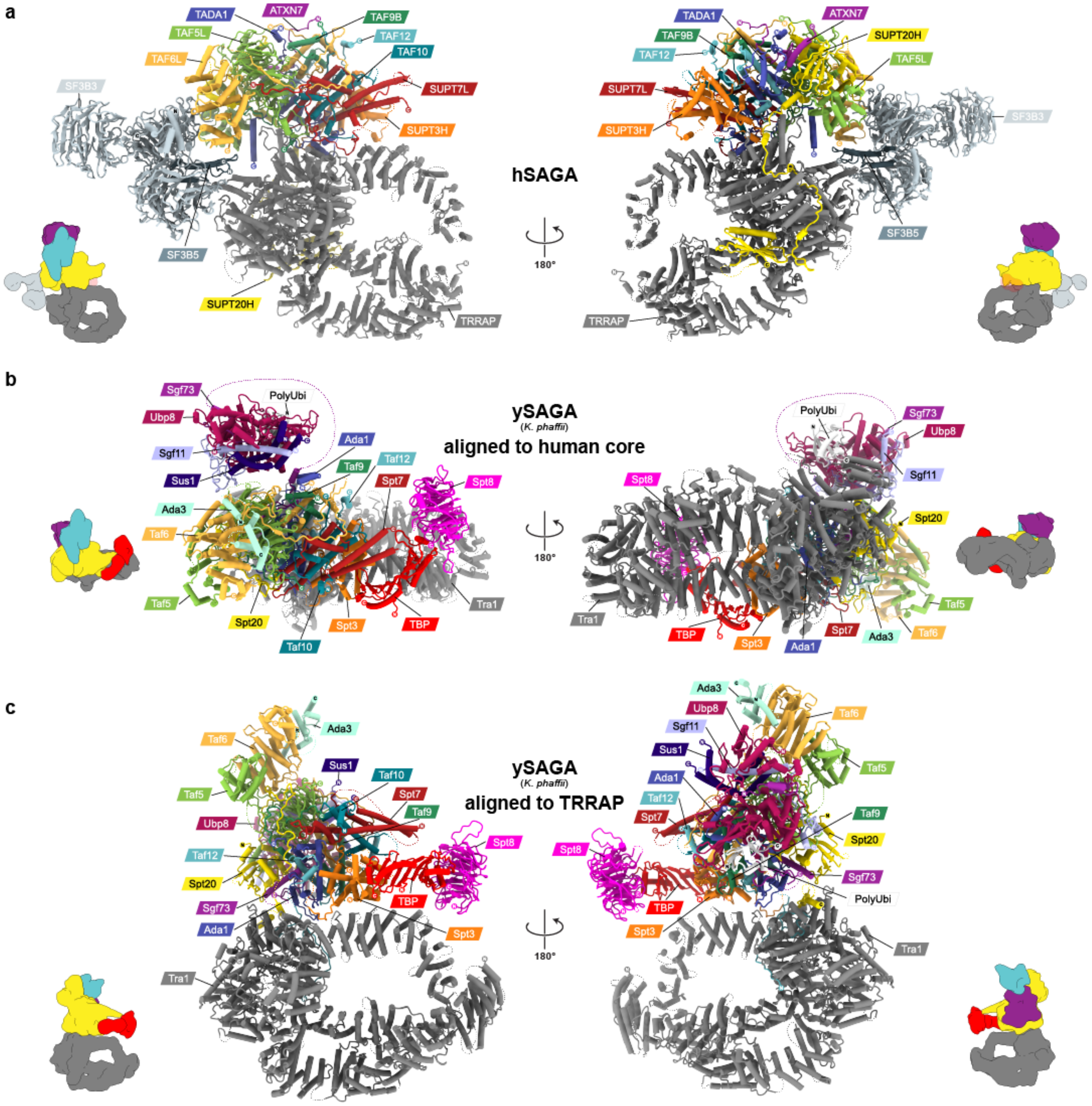


**Supplementary Figure 1 | Side-by-side comparison of (a) hSAGA (this work) and (b,c) ySAGA (PDB: 6TBM).** In (b) ySAGA is shown after superposition on the human core, aligning the WD40 propellers of TAF5/Taf5. In (c) ySAGA is shown after superposition on TRRAP, aligning residues 2323-2941 (TRRAP) and 2329-2864 (Tra1). The subunit and module coloring corresponds to Fig. 1.
