# Supplementary material for: Structure of the human SAGA coactivator complex
Source: Nat Struct Mol Biol. 2021 Nov 22;28(12):989–96. doi: 10.1038/s41594-021-00682-7 (PMC8660637; doi:10.1038/s41594-021-00682-7)
Supplement: Supplementary file 2 — Reporting Summary [file 41594_2021_682_MOESM2_ESM.pdf]

nature portfolio

Corresponding author(s): Eva Nogales

Last updated by author(s): Sep 27, 2021

# Reporting Summary

Nature Portfolio wishes to improve the reproducibility of the work that we publish. This form provides structure for consistency and transparency in reporting. For further information on Nature Portfolio policies, see our Editorial Policies and the Editorial Policy Checklist.

## Statistics

For all statistical analyses, confirm that the following items are present in the figure legend, table legend, main text, or Methods section.

| n/a | Confirmed | |
|---|---|---|
| ☐ | ☒ | The exact sample size (*n*) for each experimental group/condition, given as a discrete number and unit of measurement |
| ☒ | ☐ | A statement on whether measurements were taken from distinct samples or whether the same sample was measured repeatedly |
| ☒ | ☐ | The statistical test(s) used AND whether they are one- or two-sided<br>*Only common tests should be described solely by name; describe more complex techniques in the Methods section.* |
| ☒ | ☐ | A description of all covariates tested |
| ☒ | ☐ | A description of any assumptions or corrections, such as tests of normality and adjustment for multiple comparisons |
| ☒ | ☐ | A full description of the statistical parameters including central tendency (e.g. means) or other basic estimates (e.g. regression coefficient) AND variation (e.g. standard deviation) or associated estimates of uncertainty (e.g. confidence intervals) |
| ☒ | ☐ | For null hypothesis testing, the test statistic (e.g. $F$, $t$, $r$) with confidence intervals, effect sizes, degrees of freedom and $P$ value noted<br>*Give P values as exact values whenever suitable.* |
| ☒ | ☐ | For Bayesian analysis, information on the choice of priors and Markov chain Monte Carlo settings |
| ☒ | ☐ | For hierarchical and complex designs, identification of the appropriate level for tests and full reporting of outcomes |
| ☒ | ☐ | Estimates of effect sizes (e.g. Cohen's *d*, Pearson's *r*), indicating how they were calculated |

*Our web collection on statistics for biologists contains articles on many of the points above.*

## Software and code

Policy information about availability of computer code

| | |
|---|---|
| Data collection | SerialEM 3.7.8, Leginon 3.8.1, |
| Data analysis | Relion 3-1, CTFfind 4.1.13, LocSpiral (v. May 2020), Cryosparc 2.15.0, UCSF Chimera 1.14, , UCSF ChimeraX 2020-01-10 Rosetta 61146, Pymol 2.4.0_178, Phenix 1.18-3861 and 1.18.2-3874, Eman 2.31, Anaconda Python 2.7 and 3.7, ccp4 4-7.0, SwissModel (~June 2020), Global Phasing Limited Gelly_refine 2.10.3, Coot 0.8.9.2 and 0.9.2.0, PDBefold (fall 2020), Geneious Prime 2021.0.3, Clustal Omega 1.2.0, Al2Co first version. The custom python script cood_transform_to_star v.3.0(210604) (https://github.com/dominikaherbst/cryo-em_scripts), Sequest (Thermo Fisher Scientific, San Jose, CA, USA; version IseNode in Proteome Discoverer 1.4.1.14), Scaffold (version Scaffold_4.11.0, Proteome Software Inc.), QtPISA v2.1.0. |

For manuscripts utilizing custom algorithms or software that are central to the research but not yet described in published literature, software must be made available to editors and reviewers. We strongly encourage code deposition in a community repository (e.g. GitHub). See the Nature Portfolio guidelines for submitting code & software for further information.

## Data

Policy information about availability of data

All manuscripts must include a data availability statement. This statement should provide the following information, where applicable:

- Accession codes, unique identifiers, or web links for publicly available datasets
- A description of any restrictions on data availability
- For clinical datasets or third party data, please ensure that the statement adheres to our policy

Cryo-EM maps and refined coordinates were deposited in the Electron Microscopy Data Bank with accession codes EMD-23027 and EMD-23028 and in the Protein Data Bank with accession codes 7KTR and 7KTS.

# Field-specific reporting

Please select the one below that is the best fit for your research. If you are not sure, read the appropriate sections before making your selection.

☒ Life sciences ☐ Behavioural & social sciences ☐ Ecological, evolutionary & environmental sciences

For a reference copy of the document with all sections, see nature.com/documents/nr-reporting-summary-flat.pdf

# Life sciences study design

All studies must disclose on these points even when the disclosure is negative.

| | |
|---|---|
| Sample size | No sample size calculation was performed. Type and number of particles included in the final reconstructions were determined by resolution maximization. |
| Data exclusions | EM data processing: According to common standards in the field, not well aligning particles as well as particles representing different conformations were excluded. The particles were selected by resolution maximization. |
| Replication | EM data was collected at least three times and processing yielded similar results. |
| Randomization | Randomization was performed automatically by the single particle EM progressing software listed above. |
| Blinding | No blinding was performed as is common practice for single particle structure determination. |

# Reporting for specific materials, systems and methods

We require information from authors about some types of materials, experimental systems and methods used in many studies. Here, indicate whether each material, system or method listed is relevant to your study. If you are not sure if a list item applies to your research, read the appropriate section before selecting a response.

## Materials & experimental systems

| n/a | Involved in the study | |
|---|---|---|
| ☐ | ☒ | Antibodies |
| ☐ | ☒ | Eukaryotic cell lines |
| ☒ | ☐ | Palaeontology and archaeology |
| ☒ | ☐ | Animals and other organisms |
| ☒ | ☐ | Human research participants |
| ☒ | ☐ | Clinical data |
| ☒ | ☐ | Dual use research of concern |

## Methods

| n/a | Involved in the study | |
|---|---|---|
| ☒ | ☐ | ChIP-seq |
| ☒ | ☐ | Flow cytometry |
| ☒ | ☐ | MRI-based neuroimaging |

## Antibodies

Antibodies used

SUPT7L, Santa Cruz Biotechnology cat. # sc-514548, used in WB at 1:1000.
KAT2A, Cell Signaling Technology cat. #3305, used in WB at 1:1000.
TADA2B, Thermo Fisher Scientific cat. #PA5-57393, used in WB at 1:2500.
TBP, Abcam cat. #ab51841, used in WB at 1:2000.
USP22, Santa Cruz Biotechnology cat. #sc-390585, used in WB at 1:200.
TAF9B, #G2306, Homemade antibody previously created and validated in Herrera, Yamaguchi, Roelink & Tjian, ELife 2014. Used in WB at 1:1500.
TAF10, Millipore Sigma cat. #MABE1079, used in WB at 1:2000.
ENY2, Abcam cat. #ab183622, used in WB at 1:1000.

Validation

SUPT7L, Santa Cruz Biotechnology cat. # sc-514548: Antibody was validated by commercial supplier. From supplier: Western blotting of whole cell lysates from U-251-MG and T98G cells detect a band at ~60kDa. “SPT7L (F-5) is a mouse monoclonal antibody raised against amino acids 182-414 mapping at the C-terminus of SPT7L of human origin...SPT7L (F-5) is recommended for detection of SPT7L of mouse, rat and human origin by Western Blotting (starting dilution 1:100, dilution range 1:100-1:1000).”

KAT2A, Cell Signaling Technology cat. #3305: Antibody was validated by commercial supplier. From supplier: Western blotting of cell extracts from HeLa, NIH/3T3, C6, and COS cells show expected band at ~94kDa, and confocal immunofluorescent analysis of HeLa cells using GCN5L2 (C26A1) Rabbit mAb shows expected nuclear staining. “Monoclonal antibody is produced by immunizing animals with a synthetic peptide corresponding to the amino terminus of human GCN5L2. GCN5L2 (C26A10) Rabbit mAb detects endogenous levels of total GCN5L2 protein. The antibody does not cross-react with the related PCAF protein.”

TADA2B, Thermo Fisher Scientific cat. #PA5-57393: Antibody was validated by commercial supplier. From supplier: “Western blot analysis of TADA2B in Human cell line RT-4, U-251MG sp; Human plasma (IgG/HSA depleted), Human liver tissue, and Human tonsil

tissue. Samples were probed using a TADA2B Polyclonal Antibody. Immunohistochemical staining of TADA2B in human testis tissue shows strong nuclear positivity in cells in seminiferus ducts. Immunofluorescent staining of TADA2B in human cell line A-431 shows positivity in nucleus but excluded from the nucleoli."

TBP, Abcam cat. #ab51841: Antibody was validated by commercial supplier. From supplier: Western blot is shown for HeLa Cytoplasmic Lysate at 10ug, HeLa Nuclear Lysate at 10ug, and HeLa Nuclear Lysate at 20ug using anti-TATA binding protein TBP antibody [mAbcam 51841] - ChIP Grade (ab51841) at 5 µg/ml under reducing conditions. "This antibody gave a positive signal in the following lysates : HeLa whole cell lysate and HeLa nuclear lysate." Validation data is also shown for Immunohistochemistry, ChIP, Flow cytometry, Immunofluorescence, and Immunoprecipitation, available at supplier webpage.

USP22, Santa Cruz Biotechnology cat. #sc-390585: Antibody was validated by commercial supplier. From supplier: Western blotting of whole cell lysates from Jurkat, HeLa, Caco-2, Ca Ski, AMJ2-C8, C6, and EOC 20 cells detect a band at ~60kDa. "USP22 (C-3) is a mouse monoclonal antibody raised against amino acids 130-176 mapping within an internal region of USP22 of human origin...USP22 (C-3) is recommended for detection of USP22 of mouse, rat and human origin by Western Blotting (starting dilution 1:100, dilution range 1:100-1:1000)..."

TAF9B, #G2306, Homemade antibody previously created and validated in Herrera, Yamaguchi, Roelink & Tjian, ELife 2014.

TAF10, Millipore Sigma cat. #MABE1079: Antibody was validated by commercial supplier. From supplier: "Clone 6TA-2B11 (a.k.a. 6TA 2B11) specifically immunostained wild-type, but not Taf10-/-, mouse embryonic blastocysts (Mohan, W.S. Jr, et al.(2003). Mol .Cell. Biol. 23(12):4307-4318)... Evaluated by Western Blotting in A431 cell lysate. Western Blotting Analysis (WB): A 1:2,000 dilution of this antibody detected TAF10/TAFII30 in 10 µg of A431 cell lysate."

ENY2, Abcam cat. #ab183622: Antibody was validated by commercial supplier. From supplier: Western blotting of Jurkat whole cell lysate at 30ug with Anti-ENY2 antibody (ab183622) at 1/1000 dilution shows detection of band at ~12kDa.

## Eukaryotic cell lines

Policy information about cell lines

| | |
|---|---|
| Cell line source(s) | Cell line source(s): HeLa cells were obtained from ATCC. |
| Authentication | Provided by supplier. Endogenous knock-in SUPT7L cell line was validated using Sanger sequencing of the targeted locus +/- >500 bp on either side using multiple redundant primer pairs and Western blotting. |
| Mycoplasma contamination | Cells were routinely tested and were negative for mycoplasma contamination (microscopy fluorescence test performed by the UC Berkeley Cell Culture Facility). |
| Commonly misidentified lines (See ICLAC register) | No commonly misidentified cell lines were used in this study. |
