# Supplementary material for: Structure of the human SAGA coactivator complex
Source: Nat Struct Mol Biol. 2021 Nov 22;28(12):989–96. doi: 10.1038/s41594-021-00682-7 (PMC8660637; doi:10.1038/s41594-021-00682-7)

Source Data Extended Data Figure 1b

Uncropped scan with BSA size marker indication (from Extended Data Figure 1b). Gel was imaged with fluorescent imager on Sypro Ruby setting to visualize fluorescent Flamingo Stain.

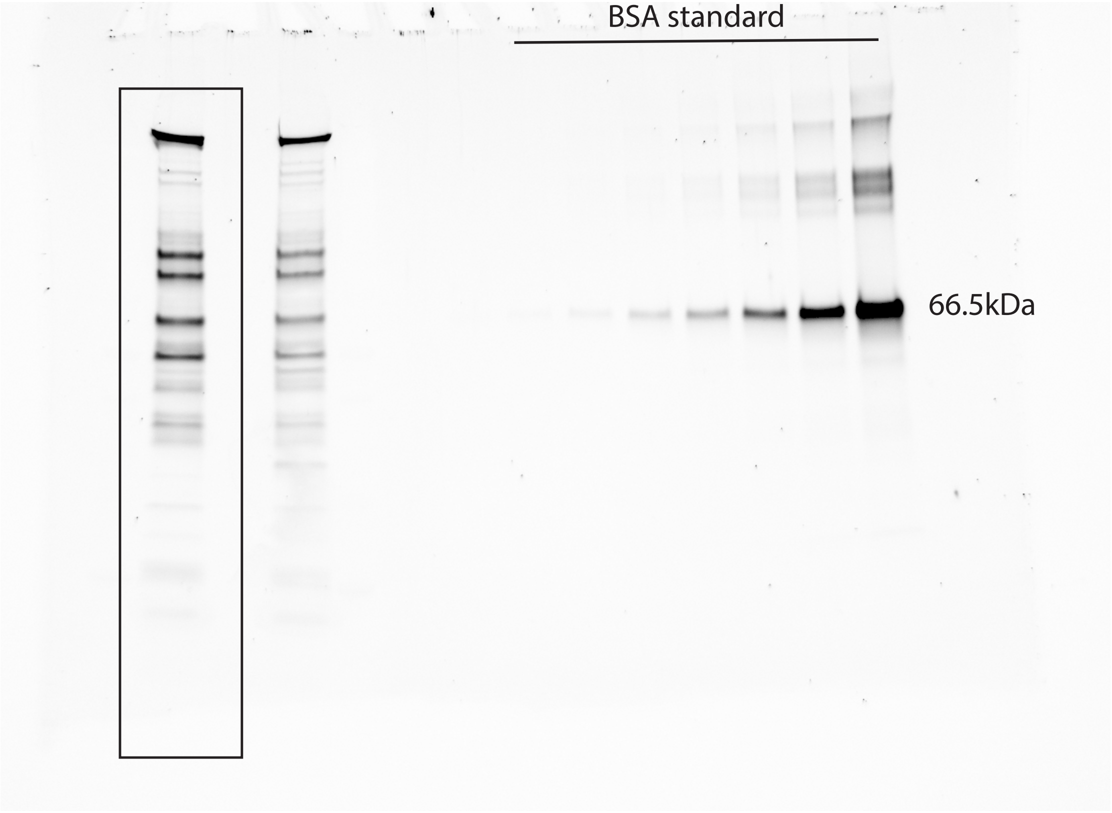

Same uncropped gel as above, imaged with white light for size marker indication.

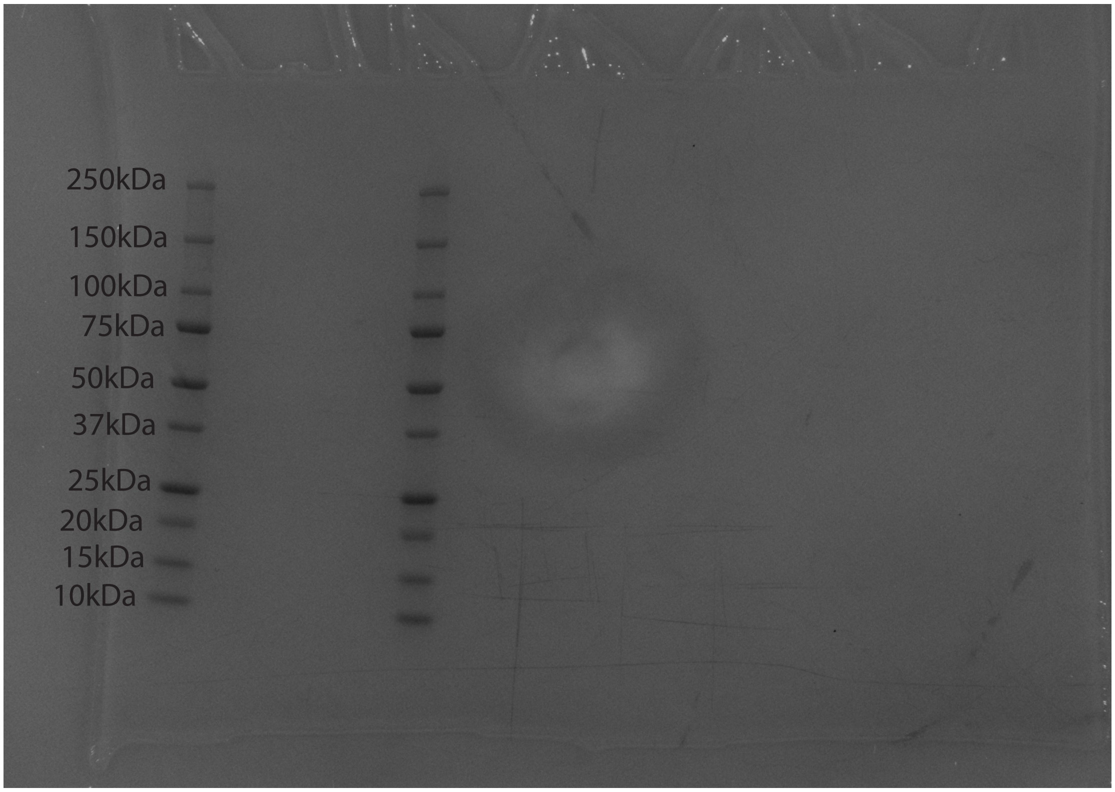

Source Data Extended Data Figure 1c

Uncropped Western blot imaged with chemiluminescence (from Extended Data Figure 1c).
Cropped regions from Extended Data Fig. 1c are highlighted with black rectangles.

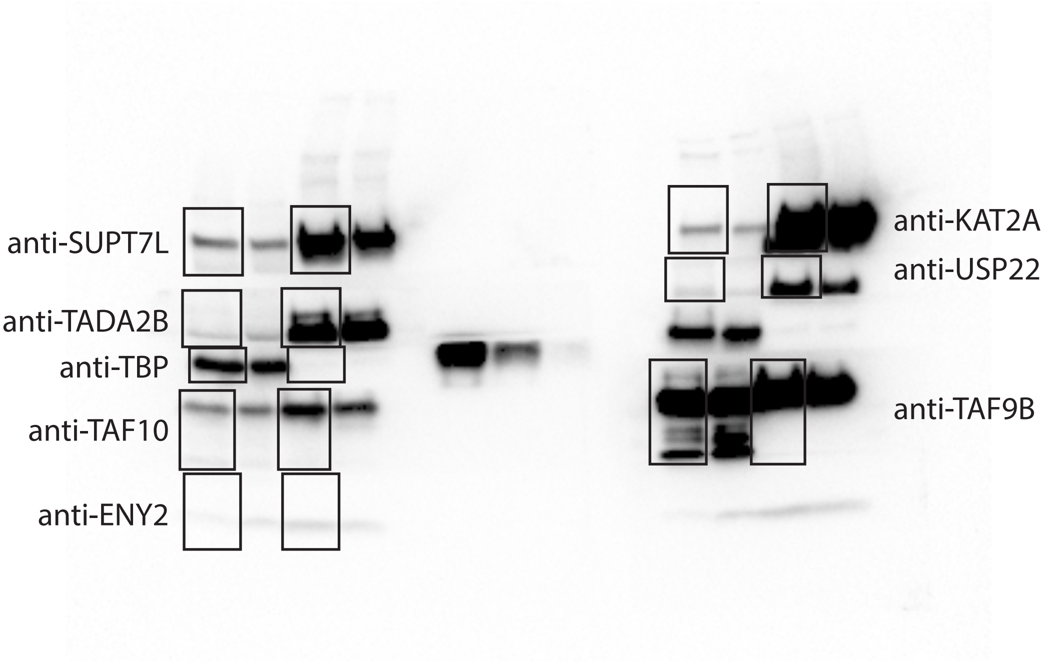

Same uncropped scan as above, imaged with white light with size marker indication.

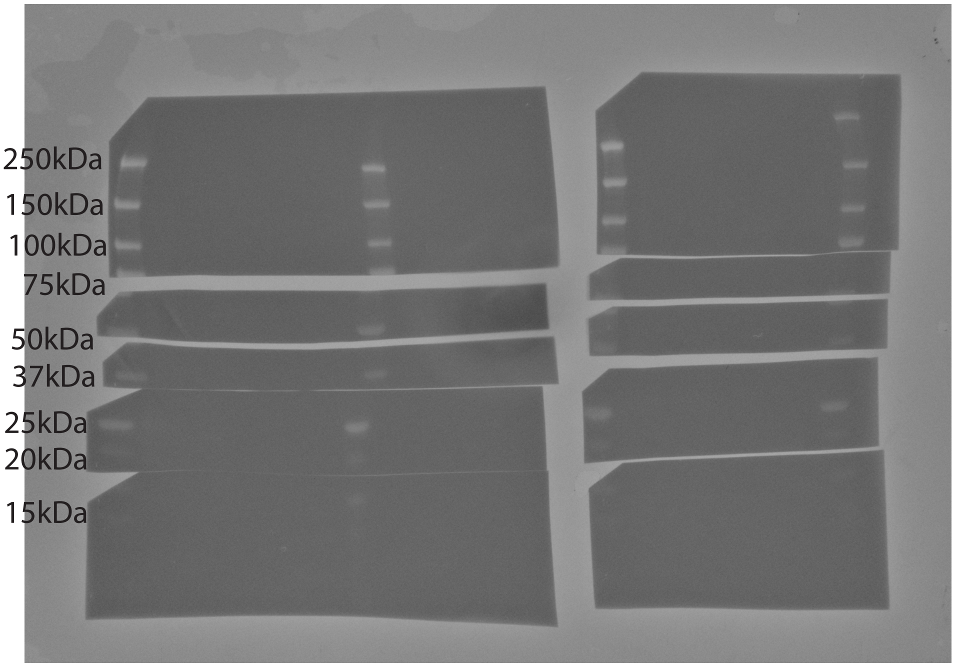

Supplement: Source Data Extended Data Fig. 1 — Uncropped scan with size marker indication (from Extended Data Fig. 1b) and uncropped western blot imaged with chemiluminescence (from Extended Data Fig. 1c). [file 41594_2021_682_MOESM4_ESM.pdf]
